# Preventive use of respiratory support after scheduled extubation in critically ill medical patients—a network meta-analysis of randomized controlled trials

**DOI:** 10.1186/s13054-020-03090-3

**Published:** 2020-06-22

**Authors:** Xiaoyang Zhou, Shengmi Yao, Pingping Dong, Bixin Chen, Zhaojun Xu, Hua Wang

**Affiliations:** 1 Department of Intensive Care Medicine, HwaMei Hospital, University of Chinese Academy of Sciences, Ningbo, 315000 Zhejiang China; 2 Ningbo Institute of Life and Health Industry, University of Chinese Academy of Sciences, Ningbo, 315000 Zhejiang China; 3 Emergency intensive care unit, Ningbo Yinzhou No.2 Hospital, Ningbo, 315000 Zhejiang China; 4 Baihe Street Community Health Services of Yinzhou District, Ningbo, 315000 Zhejiang China

**Keywords:** Noninvasive ventilation, High-flow oxygen therapy, Tracheal extubation, Re-intubation, Mortality, Network meta-analysis

## Abstract

**Background:**

Respiratory support has been increasingly used after extubation for the prevention of re-intubation and improvement of prognosis in critically ill medical patients. However, the optimal respiratory support method is still under debate. This network meta-analysis (NMA) aims to evaluate the comparative effectiveness of various respiratory support methods used for preventive purposes after scheduled extubation in critically ill medical patients.

**Methods:**

A systematic database search was performed from inception to December 19, 2019, for randomized controlled trials (RCTs) that compared a preventive use of different respiratory support methods, including conventional oxygen therapy (COT), noninvasive ventilation (NIV), high-flow oxygen therapy (HFOT), and combinational use of HFOT and NIV (HFOT+NIV), after planned extubation in adult critically ill medical patients. Study selection, data extraction, and quality assessments were performed in duplicate. The primary outcomes included re-intubation rate and short-term mortality.

**Results:**

Seventeen RCTs comprising 3341 participants with 4 comparisons were included. Compared with COT, NIV significantly reduced the re-intubation rate [risk ratio (RR) 0.55, 95% confidence interval (CI) 0.39 to 0.77; moderate quality of evidence] and short-term mortality (RR 0.66, 95% CI 0.48 to 0.91; moderate quality of evidence). Compared to COT, HFOT had a beneficial effect on the re-intubation rate (RR 0.55, 95% CI 0.35 to 0.86; moderate quality of evidence) but no effect on short-term mortality (RR 0.79, 95% CI 0.56 to 1.12; low quality of evidence). No significant difference in the re-intubation rate or short-term mortality was found among NIV, HFOT, and HFOT+NIV. The treatment rankings based on the surface under the cumulative ranking curve (SUCRA) from best to worst for re-intubation rate were HFOT+NIV (95.1%), NIV (53.4%), HFOT (51.2%), and COT (0.3%), and the rankings for short-term mortality were NIV (91.0%), HFOT (54.3%), HFOT+NIV (43.7%), and COT (11.1%). Sensitivity analyses of trials with a high risk of extubation failure for the primary outcomes indicated that the SUCRA rankings were comparable to those of the primary analysis.

**Conclusions:**

After scheduled extubation, the preventive use of NIV is probably the most effective respiratory support method for comprehensively preventing re-intubation and short-term death in critically ill medical patients, especially those with a high risk of extubation failure.

## Background

Invasive mechanical ventilation (IMV) is universally recognized as a first-line therapy for rescuing acute respiratory failure. Although it is a life-saving treatment in nature, prolonged IMV is always accompanied by an increased risk of ventilator-associated pneumonia and lung injury [1, 2] and neurocognitive sequelae associated with prolonged sedation [3, 4], thus resulting in a longer duration of intensive care unit (ICU) stay and increased mortality [5, 6]. Therefore, it is essential for mechanically ventilated patients to receive daily assessments of weaning readiness [6] and timely extubation when they meet the criteria of weaning from IMV. However, approximately 10–20% of patients will experience extubation failure and require re-intubation within 24–72 h after scheduled extubation [7–10], and extubation failure is associated with poor outcomes and increased mortality [8]. It is therefore essential to receive prophylactic respiratory support for post-extubated patients.

Conventional oxygen therapy (COT) is the most frequently administered respiratory support method after extubation. COT can only deliver a maximum flow of oxygen (O_2_) of 15 L/min using the Venturi mask or reservoir mask [11], and the delivered fraction of inspired oxygen (FiO_2_) is unstable because the FiO_2_ also depends on the inspiratory flow, respiration rate, and tidal volume of patients in addition to the O_2_ flow [12]. Hence, apart from improving oxygenation, COT seems to have no or minimal effects on changes in lung aeration, hemodynamics, or neuromuscular function, which are the main pathophysiological mechanisms that contributed to extubation failure [12]. In recent years, noninvasive ventilation (NIV) and high-flow oxygen therapy (HFOT) have been increasingly used as alternative respiratory support methods in post-extubated patients. Both NIV and HFOT are anticipated to prevent extubation failure and improve prognosis by delivering more stable FiO_2_ [12, 13], promoting alveolar recruitment and preventing alveolar collapse [14–16], and reducing the work of breathing [17, 18].

Nevertheless, the latest meta-analysis [19] of randomized controlled trials (RCTs) suggested that compared to COT, preventive use of NIV after extubation had no effect on the re-intubation rate or mortality in post-extubated patients. Meanwhile, several recent meta-analyses [20–22] also revealed neutral effects of HFOT used after planned extubation on the re-intubation rate or mortality compared with COT or NIV. More recently, a multicenter RCT [23] proposed a novel method that combined the use of HFOT and NIV (HFOT+NIV) and proved its superiority over HFOT in the prevention of re-intubation in post-extubated patients. However, the method did not affect mortality. Although the above studies are informative, the relative effectiveness throughout various respiratory support methods remains unknown. Unlike conventional pairwise meta-analysis that only include head-to-head comparisons, network meta-analysis (NMA) can compare multiple treatments simultaneously in a single analysis by combining direct and indirect evidence [24] and inform on the relative effect of indirectly compared treatments. Therefore, we conducted an NMA to evaluate the comparative effectiveness of various respiratory support methods used as a preventive strategy after planned extubation in critically ill medical patients.

## Methods

This NMA was performed in accordance with the Preferred Reporting Items for Systematic Reviews and Meta-Analyses (PRISMA) Extension statement for reporting network meta-analyses [25]. The study protocol was registered at the international prospective register of systematic reviews (PROSPERO registration number: CRD42020164357).

### Search strategy

Relevant studies regarding preventive use of various respiratory support methods, including COT, NIV, HFOT, and HFOT+NIV, after planned extubation in critically ill medical patients were searched systematically by two independent reviewers (Xu Z and Chen B) from database inception through December 19, 2019, in PubMed, Embase, Web of Science, and Cochrane Central Register of Controlled Trials. The detailed search strategy is presented in Additional file 1. A manual search of reference lists from previous relevant studies and reviews was also conducted to further identify relevant literature. This NMA had no restrictions on language or date of publications.

### Study selection

After filtering duplicate records, two reviewers (Xu Z and Chen B) independently screened the title and abstract for eligibility. The full text of records deemed eligible during preliminary screening was reviewed to determine whether these studies met the inclusion or exclusion criteria. The reasons for the exclusion of irrelevant studies are recorded in Additional file 1 (Table S1). No restrictions were applied on study period, primary disease leading to IMV, ventilation mode in NIV, or risk of extubation failure. A third reviewer participated in the discussion to adjudicate disagreements.

### Criteria for inclusion and exclusion

The inclusion criteria included the following: (1) participants: adult critically ill medical patients (age ≥ 18 years) admitted to the ICU who received IMV > 12 h, successfully passed the spontaneous breathing trial (SBT), and were ready for extubation; (2) interventions and comparisons: one of the following respiratory support methods compared with one another: COT, NIV, HFOT, and HFOT+NIV. All of these methods were used after planned extubation for preventive purposes; (3) outcomes: the primary outcomes were re-intubation rate and short-term mortality, and the secondary outcomes included post-extubation respiratory failure, length of ICU stay and in-hospital stay, and comfort score. Studies reporting on at least one of the above outcomes were included. The short-term mortality was predefined as death within 30 days after randomization irrespective of the cause of death; and (4) study design: prospective RCTs.

The exclusion criteria included the following: (1) non-RCTs, including reviews, retrospective studies, cohort studies, and crossover studies; (2) studies conducted in post-surgical patients; (3) studies enrolled patients who underwent an unplanned extubation; (4) studies in which respiratory support was used for therapeutic or facilitative purpose [12]; (5) studies did not report any outcomes of interest; and (6) conference abstracts without full-text manuscripts.

### Data extraction

Two reviewers (Yao S and Dong P) independently reviewed the complete text of each included study and extracted data using a standardized form. The abstracted data included the name of the first author, publication year, sample size, details of the population enrolled, primary diagnosis leading to IMV, characteristics of interventions, study period, acute physiology and chronic health evaluation (APACHE) II score, and atrial partial pressure of carbon dioxide (PaCO_2_) at end of SBT. Data on primary and secondary outcomes were also recorded in detail. If a study reported various mortalities, the longest follow-up short-term mortality was used for analysis. Data on the occurrence of re-intubation and respiratory failure within 72 h after extubation was preferred, and it would, if unavailable, be substituted by data on occurrence during ICU admission. We also used the PaCO_2_ measured during SBT or at extubation instead of that measured at the end of SBT when it was unavailable. The disagreement was resolved by a joint review of the full text to reach consensus.

The criteria for diagnosing post-extubation respiratory failure were defined by the authors in the included trials. According to the previous studies [23, 26–28], we predefined “high risk” of extubation failure as the presence of at least one among the following factors: (1) age > 65 years; (2) heart failure or chronic obstructive pulmonary disease (COPD); (4) APACHE II score > 12 at extubation; (5) body mass index > 30 kg/m^2^; (6) airway patency problems, including high risk of developing laryngeal edema or inability to deal with respiratory secretions; (7) 2 or more comorbidities; (8) more than one SBT failure; and (9) IMV > 7 days.

### Quality assessment

Two independent reviewers (Yao S and Dong P) evaluated the quality of each included trials using the Cochrane risk of bias tool [29]. Each trial was judged as low, unclear, or high risk with respect to adequate sequence generation, allocation concealment, blinding of participants and personnel, blinding of outcome assessment, incomplete outcome data, selective reporting, and other bias. We resolved disagreements by a discussion with a third reviewer to reach consensus.

### Statistical analysis

The random effects NMA was performed using a frequentist framework to calculate risk ratios (RR) for dichotomous outcomes, mean differences (MD) for continuous outcomes, and corresponding 95% confidence intervals (CI). The conventional pairwise meta-analyses were also conducted for each comparison using a random effects model. All statistical analyses were performed using the netmeta package in Stata/SE 15.0 (Stata-Corp, College Station, TX, USA). Two-sided *P* value less than 0.05 was considered statistically significant.

Homogeneity and consistency assumptions underlie the validity of evidence from NMA [30]. To evaluate heterogeneity across studies within each direct comparison, we visually inspected the forest plots and quantified using the *Q* test and the *I*^2^ statistic [31]. Inconsistency between direct and indirect estimates in the entire network for each outcome was assessed locally with a loop-specific approach and globally with design-by-treatment interaction model [32]. We also ranked the treatment effects of various respiratory support methods according to the probabilities of leading to the best results based on the surface under the cumulative ranking curve (SUCRA) for each outcome [33]. The value of SCURA ranges from 0 to 100%, the higher the value, the better the effectiveness of the method [33].

Given that the risk of extubation failure and hypercapnia (PaCO_2_ > 45 mmHg) at the end of SBT might affect the relative effectiveness of various respiratory support methods [34], we performed two sensitivity analyses to evaluate the robustness of the NMA results for the primary outcomes by excluding studies with low or unclear risk of extubation failure or studies that enrolled patients with hypercapnia at the end of SBT.

### Grading the quality of evidence

We assessed the quality of evidence from direct comparisons, indirect comparisons, and NMA estimates for each outcome using the modified Grading of Recommendation, Assessment, Development and Evaluation (GRADE) tool for NMA [35, 36]. The contribution matrix was constructed to evaluate the information contribution of direct evidence to entire NMA estimates [36]. Because only one loop (NIV-COT-HFOT) was connected in this NMA, we assigned the quality of the indirect comparison with the lower quality rating in the two contributing direct comparisons within this loop. Additionally, the higher confidence in the direct and indirect estimates was preferred as the quality rating of overall NMA estimates. The quality of evidence would be rated down for the presence of risk of bias, imprecision, publication bias, indirectness, intransitivity, or incoherence between direct and indirect estimates [36].

## Results

### Study selection

**Fig. 1:**
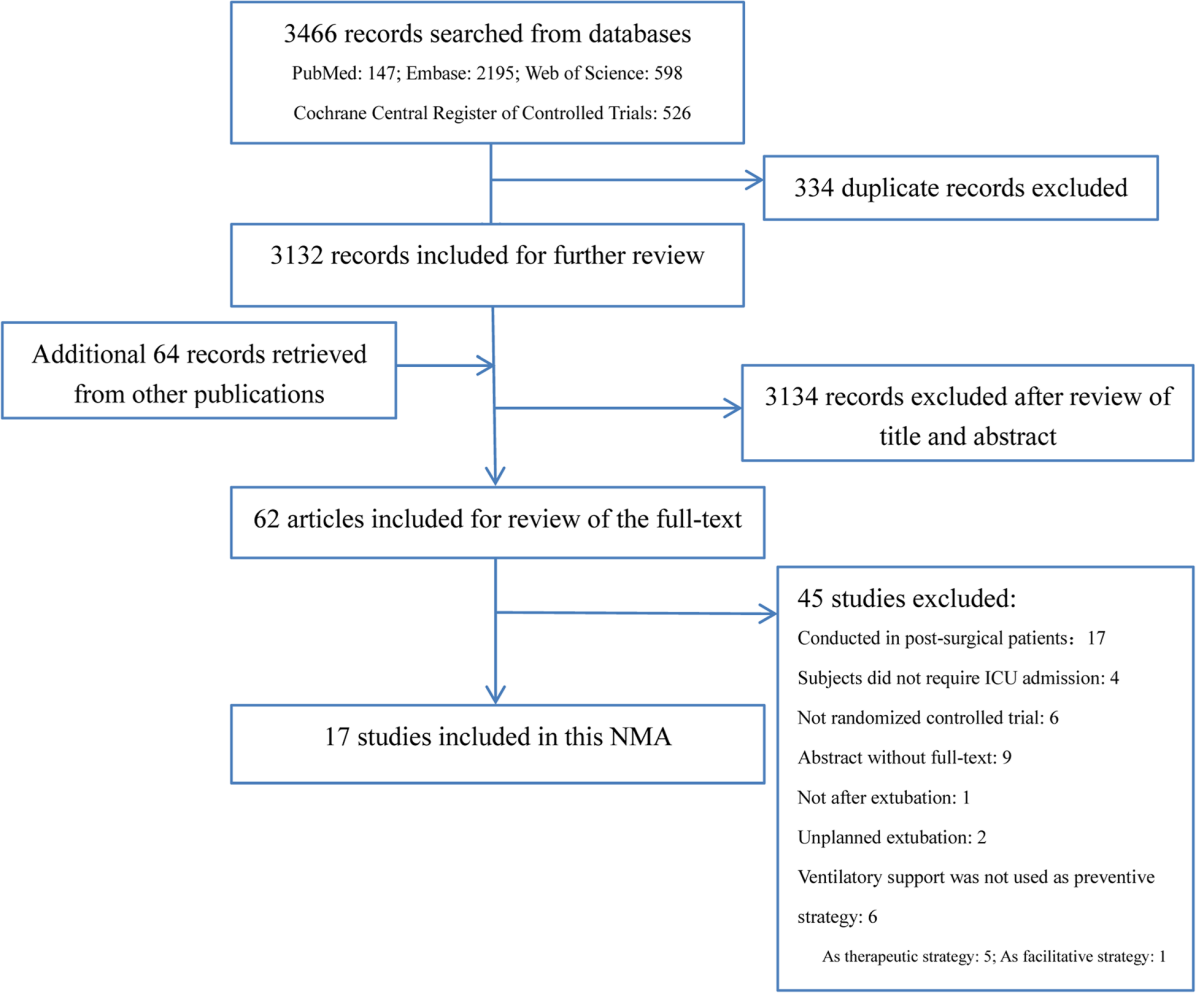
PRISMA flowchart for the study selection process

We initially identified 3466 citations through the electronic database search. An additional 64 records were identified from the manual search of the references in previous publications. After excluding 334 duplicates and 3134 irrelevant citations, we reviewed the full text of the remaining 62 records. Finally, a total of 17 eligible RCTs [23, 26–28, 37–49], representing 3341 patients, were included in this NMA. The PRISMA flowchart for study inclusion is shown in Fig. 1.

### Study characteristics

Of the 17 included RCTs [23, 26–28, 37–49], 10 [23, 26–28, 38, 41, 43–46] were multicenter, and 7 [37, 39, 40, 42, 47–49] were single-center. All included RCTs were published in the last 15 years, and the number of participants ranged from 38 to 614. Participants in 5 trials [23, 26–28, 41] were at high risk of extubation failure, and participants in 1 trial [45] were at low risk. The specified definition of risk of extubation failure was unavailable in 11 trials [37–40, 42–44, 46–49]. Among these trials, however, 9 trials [37–40, 42, 44, 47–49] fulfilled the predefined criteria of a high risk of extubation failure in our NMA and were therefore classified as high risk, and the risk in the remaining 2 trials [43, 46] was unclear. NIV was compared with COT in 9 trials [26, 37–44]. Four trials compared HFOT with COT [28, 45–47]. Three trials compared NIV with HFOT [27, 48, 49], and 1 trial [23] compared the combinational use of HFOT and NIV (HFOT+NIV) with HFOT alone. In all trials that involved NIV [23, 26, 27, 37–44, 48, 49], NIV was used with bilevel positive airway pressure mode. The PaCO_2_ level at the end of SBT was less than 45 mmHg in 11 trials [23, 26–28, 40–42, 45–47, 49], greater than 45 mmHg in 4 trials [38, 39, 44, 48], and unavailable in 2 trials [37, 43]. Details regarding the characteristics and outcomes of each included study are described in Additional file 1 (Table S2 and S3).

### Risk of bias

**Fig. 2:**
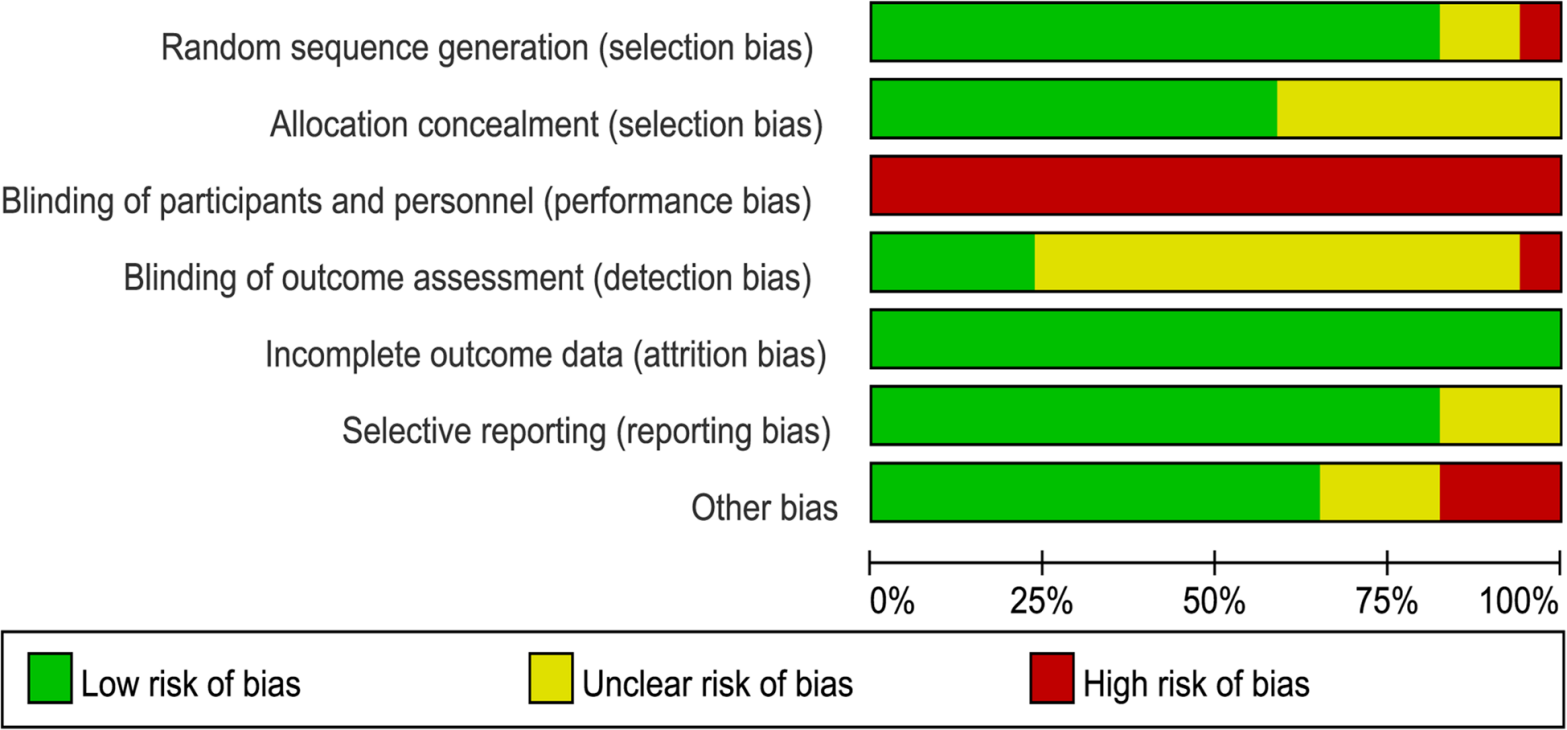
Risk of bias graph. Reviewers’ judgments about each risk of bias item presented as percentages across all included studies

**Fig. 3:**
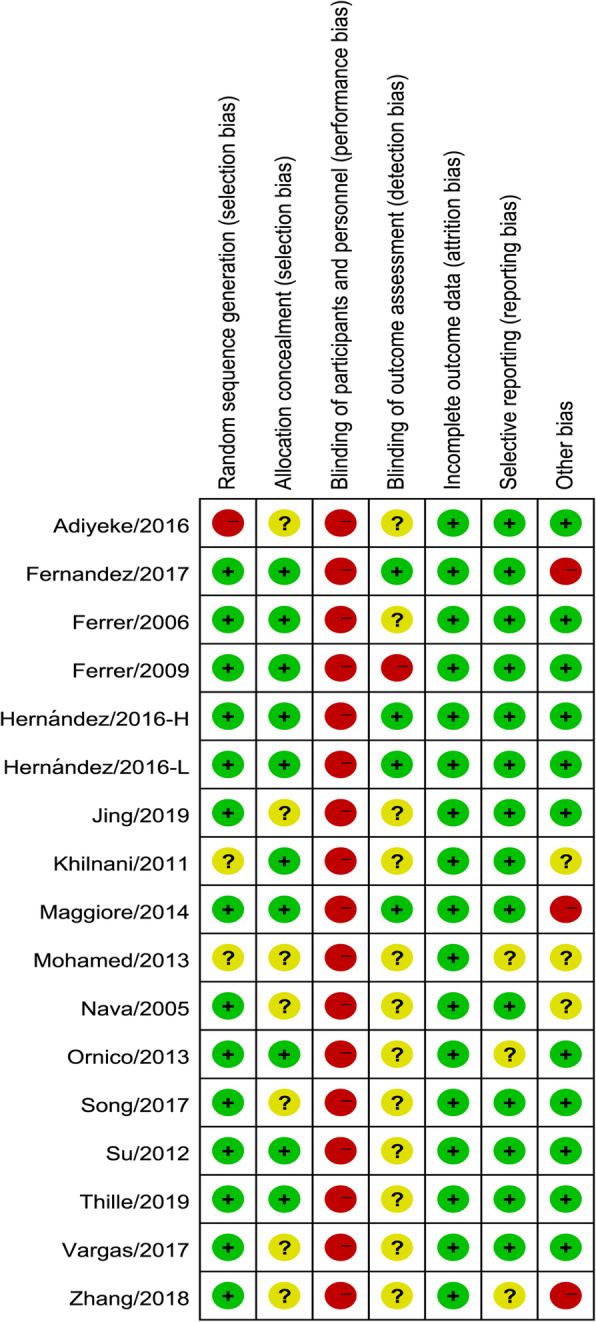
Risk of bias summary. Reviewers’ judgments about each risk of bias item for each included study

The quality assessment is presented in detail in Figs. 2 and 3. All trials were assessed to be at low or unclear risk of bias in terms of adequate sequence generation and allocation concealment except for one trial [37] in which randomization was performed based on the admission number. Of note, all trials were judged as having a high risk of bias in blinding of participants and personnel because it was clinically impracticable due to virtual practice issues. Apart from one trial [38] that had a high bias in detection, all other trials had a low or unclear risk of bias in detection, attrition, and reporting. Additionally, three trials [28, 46, 49] had a high risk of other bias associated with the funding source.

### Quality of evidence

We downgraded the quality of evidence for several direct comparisons due to imprecision, limitations of risk of bias, or statistical heterogeneity. We had no significant concerns on intransitivity. Although no statistical evidence of incoherency was found in the network for any outcomes, we downgraded the quality of evidence for the length of ICU stay and length of in-hospital stay in two comparisons due to the presence of problematic incoherence that was evaluated by visually inspecting the direct and indirect estimates. The summary of evidence grading is presented in Table 1.

**Table 1:** Quality assessment and treatment effect estimates from conventional meta-analysis and network meta-analysis for each outcome

Comparisons	No. of RCTs	***I***^**2**^ value	Pooled result from CMA	Direct estimate	Quality	Indirect estimate	Quality^**5**^	NMA estimate	Quality
Re-intubation rate (RR with 95%CI); test for inconsistency in the entire network: *P* = 0.214									
NIV vs. COT	9	19.8%	0.62 (0.46, 0.83)	0.60 (0.42, 0.87)	Moderate^1^	0.35 (0.16, 0.78)	Low^6,7^	0.55 (0.39, 0.77)	Moderate^11^
HFOT vs. COT	4	7.7%	0.45 (0.27, 0.73)	0.44 (0.25, 0.77)	Moderate^1^	0.76 (0.40, 1.47)	Low^6,7^	0.55 (0.35, 0.86)	Moderate^11^
NIV vs. HFOT	3	0.0%	0.82 (0.61, 1.12)	0.79 (0.45, 1.39)	Low^1,2^	1.37 (0.71, 2.63)	Moderate^6^	1.00 (0.64, 1.54)	Moderate^11^
HFOT+NIV vs. HFOT	1	NE	0.57 (0.37, 0.87)	0.57 (0.29, 1.12)	Moderate^1^	NE^8^		0.57 (0.29, 1.12)	Moderate^11^
Short-term mortality (RR with 95%CI); test for inconsistency in the entire network: *P* = 0.355									
NIV vs. COT	9	0.0%	0.60 (0.41, 0.87)	0.60 (0.41, 0.87)	Moderate^1^	0.83 (0.46, 1.48)	Low^6,7^	0.66 (0.48, 0.91)	Moderate^11^
HFOT vs. COT	3	0.0%	0.93 (0.57, 1.52)	0.93 (0.57, 1.52)	Low^1,2^	0.67 (0.41, 1.09)	Low^6,7^	0.79 (0.56, 1.12)	Low^11,12^
NIV vs. HFOT	3	0.0%	0.89 (0.65, 1.22)	0.89 (0.65, 1.22)	Low^1,2^	0.64 (0.35, 1.19)	Low^6,7^	0.84 (0.63, 1.10)	Low^11,12^
HFOT+NIV vs. HFOT	1	NE	1.05 (0.73, 1.50)	1.05 (0.73, 1.50)	Low^1,2^	NE^8^		1.05 (0.73, 1.50)	Low^11,12^
Post-extubation respiratory failure (RR with 95%CI); test for inconsistency in the entire network: *P* = 0.684									
NIV vs. COT	5	78.0%^a^	0.42 (0.22, 0.81)	0.43 (0.23, 0.78)	Low^1,3^	0.57 (0.15, 2.16)	Low^6,7^	0.45 (0.27, 0.78)	Low^11,12^
HFOT vs. COT	3	55.0%	0.52 (0.30, 0.92)	0.50 (0.23, 1.07)	Moderate^1^	0.37 (0.11, 1.23)	Low^6,7^	0.46 (0.25, 0.84)	Moderate^11^
NIV vs. HFOT	2	36.0%	1.14 (0.39, 3.32)	1.15 (0.40, 3.35)	Low^1,2^	0.86 (0.32, 2.25)	Low^6,9^	0.99 (0.50, 1.97)	Low^11,12^
HFOT+NIV vs. HFOT	1	NE	0.71 (0.54, 0.93)	0.71 (0.24, 2.09)	Moderate^1^	NE^8^		0.71 (0.24, 2.09)	Moderate^11^
Length of ICU stay (MD with 95%CI); test for inconsistency in the entire network: *P* = 0.255									
NIV vs. COT	8	90.7%^a^	− 2.18 (− 4.45, 0.09)	− 2.17 (− 4.43, 0.08)	Very Low^1,2,3^	0.91 (− 3.90, 5.72)	Low^6,7^	− 1.62 (− 3.68, 0.44)	Very Low^11,12,13^
HFOT vs. COT	3	0.0%	− 0.05 (− 0.83, 0.72)	− 0.24 (− 3.76, 3.28)	Low^1,2^	− 3.32 (− 7.30, 0.66)	Very Low^6,7,9^	− 1.59 (− 4.25, 1.06)	Low^11,12^
NIV vs. HFOT	3	0.0%	1.37 (1.03, 1.72)	1.15 (− 2.13, 4.42)	Moderate^1^	− 1.94 (− 6.12, 2.25)	Very Low^6,7,9^	−0.03 (− 2.63, 2.58)	Low^11,13^
HFOT+NIV vs. HFOT	1	NE	1.00 (− 0.38, 2.38)	1.00 (− 4.70, 6.70)	Low^1,2^	NE^8^		1.00 (−4.70, 6.70)	Low^11,12^
Length of in-hospital stay (MD with 95%CI); test for inconsistency in the entire network: *P* = 0.280									
NIV vs. COT	4	0.0%	− 0.52 (− 3.58, 2.55)	− 0.52 (− 3.58, 2.55)	Low^1,2^	2.02 (− 1.42, 5.46)	Very Low^6,10^	0.61 (−1.68, 2.89)	Very Low^11,12,13^
HFOT vs. COT	2	0.0%	− 0.98 (− 2.17, 0.22)	− 0.98 (− 2.17, 0.22)	Low^1,2^	− 3.52 (− 7.97, 0.93)	Very Low^6,10^	−1.15 (− 2.30, 0.00)	Low^11,12^
NIV vs. HFOT	1	NE	3.00 (− 0.23, 6.23)	3.00 (− 0.23, 6.23)	Very low^1,4^	0.46 (− 2.83, 3.75)	Low^6,7^	1.75 (−0.55, 4.06)	Low^11,12^
HFOT+NIV vs. HFOT	1	NE	2.00 (− 0.93, 4.93)	2.00 (− 0.93, 4.93)	Low^1,2^	NE^8^		2.00 (−0.93, 4.93)	Low^11,12^
Comfort score (MD with 95%CI)									
HFOT vs. COT	2	0%	− 1.96 (− 2.44, − 1.49)						
NIV vs. HFOT	1	NE	1.60 (0.32, 2.88)						

Both CMA and NMA were performed using the random effect model. Numbers in parentheses are the 95% CI

*No.* number, *RCTs* randomized controlled trials, *CMA* conventional meta-analysis, *NMA* network meta-analysis, *NIV* noninvasive ventilation, *HFOT* high-flow oxygen therapy, *COT* conventional oxygen therapy, *ICU* intensive care unit, *RR* risk ratio, *MD* mean difference, *CI* confidence interval, *NE* not estimable

^a^*P* < 0.05

^1^Quality of evidence for direct estimate rated down by one level for serious risk of bias because of the high risk of unblinding of participants and personnel in all included trials

^2^Quality of evidence for direct estimate rated down by one level for serious imprecision because 95% CI include values favoring either treatment

^3^Quality of evidence for direct estimate rated down by one level for substantial heterogeneity

^4^Quality of evidence for direct estimate rated down by two levels for very serious imprecision because 95% CI are very wide and include values favoring either treatment

^5^Quality of evidence will be not downgraded for intransitivity in the indirect comparisons

^6^Quality of evidence for indirect estimate rated down by one level for serious risk of bias

^7^Quality of evidence for indirect estimate rated down by one level for serious imprecision

^8^Not estimable because no loop can be constructed for the two treatments in the evidence network

^9^Quality of evidence for indirect estimate rated down by one level for serious incoherence

^10^Quality of evidence for indirect estimate rated down by two levels for very serious imprecision

^11^Quality of evidence for network estimate rated down by one level for serious risk of bias

^12^Quality of evidence for network estimate rated down by one level for serious imprecision

^13^Quality of evidence for network estimate rated down by one level for potential serious incoherence

### Analysis of the primary outcomes

**Fig. 4:**
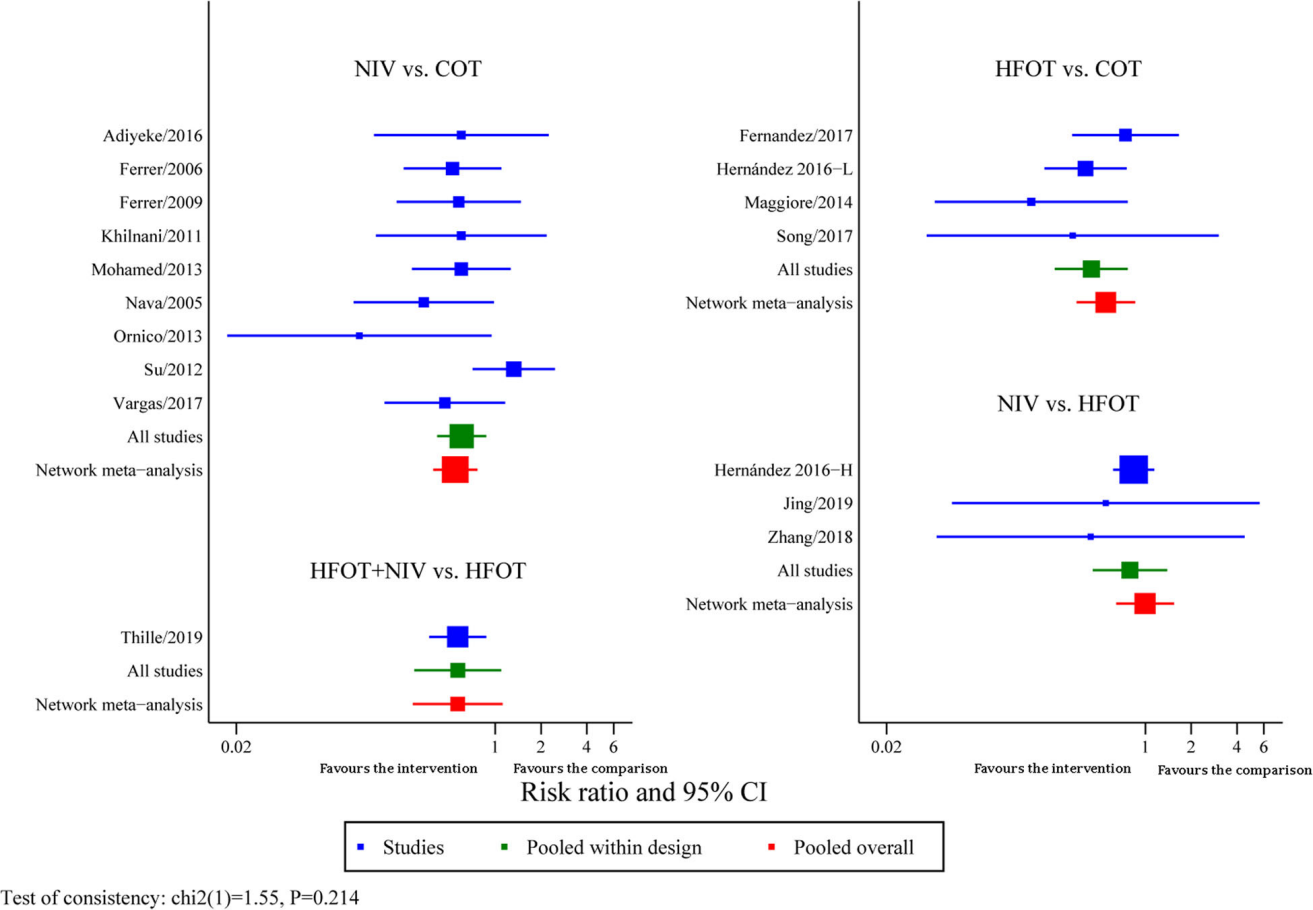
Forest plot of network meta-analysis for re-intubation rate. NIV noninvasive ventilation, HFOT high-flow oxygen therapy, COT conventional oxygen therapy, CI confidence interval

All included RCTs [23, 26–28, 37–49] involving 3341 patients reported re-intubation rates. No statistically significant heterogeneity was noted among the included trials within each comparison (Table 1). The inconsistency test at the global and local levels indicated no significant inconsistency (Fig. 4, Fig. S1 in Additional file 1). The quality of evidence for NMA estimates was rated as moderate (Table 1). Compared with COT, NIV and HFOT were similarly effective in reducing the re-intubation rate (RR 0.55, 95% CI 0.39 to 0.77 and RR 0.55, 95% CI 0.35 to 0.86, respectively) (Fig. 4). Indirect evidence showed that compared to NIV or HFOT, HFOT+NIV likely decreased the re-intubation rate (Table 2) despite the lack of statistical significance. Thus, HFOT+NIV ranked best according to the SUCRA statistic followed by NIV, HFOT, and COT (Table 3).

**Table 2:** League table for networks of respiratory support methods

Re-intubation rate (RR with 95% CI)			
**COT**	0.55 (0.35, 0.86)	0.55 (0.39, 0.77)	0.31 (0.14, 0.70)
	**HFOT**	1.00 (0.64, 1.54)	0.57 (0.29, 1.12)
		**NIV**	0.57 (0.26, 1.28)
			**HFOT + NIV**
Short-term mortality (RR with 95% CI)			
**COT**	0.79 (0.56, 1.12)	0.66 (0.48, 0.91)	0.83 (0.50, 1.36)
	**HFOT**	0.84 (0.63, 1.10)	1.05 (0.73, 1.50)
		**NIV**	1.25 (0.79, 1.98)
			**HFOT + NIV**
Post-extubation respiratory failure (RR with 95% CI)			
**COT**	0.46 (0.25, 0.84)	0.45 (0.27, 0.78)	0.32 (0.09, 1.12)
	**HFOT**	0.99 (0.50, 1.97)	0.71 (0.24, 2.09)
		**NIV**	0.71 (0.20, 2.56)
			**HFOT + NIV**
Length of ICU stay (MD with 95%CI)			
**COT**	− 1.59 (− 4.25, 1.06)	− 1.62 (− 3.68, 0.44)	− 0.59 (− 6.88, 5.69)
	**HFOT**	− 0.03 (− 2.63, 2.58)	1.00 (− 4.70, 6.70)
		**NIV**	1.03 (− 5.24, 7.30)
			**HFOT + NIV**
Length of in-hospital stay (MD with 95%CI)			
**COT**	− 1.15 (− 2.30, 0.00)	0.61 (− 1.68, 2.89)	0.85 (− 2.29, 4.00)
	**HFOT**	1.75 (− 0.55, 4.06)	2.00 (− 0.93, 4.93)
		**NIV**	0.25 (− 3.48, 3.97)
			**HFOT + NIV**

The column treatment is compared with the row treatment. Numbers in parentheses are the 95% CI

*COT* conventional oxygen therapy, *NIV* noninvasive ventilation, *HFOT* high-flow oxygen therapy, *ICU* intensive care unit, *RR* risk ratio, *MD* mean difference, *CI* confidence interval

**Table 3:** SCURA statistics for each outcome

Outcomes	COT (%)	NIV (%)	HFOT (%)	HFOT + NIV (%)
Re-intubation rate	0.3	53.4	51.2	95.1
Short-term mortality	11.1	91.0	54.3	43.7
Post-extubation respiratory failure	1.6	60.6	58.6	79.2
Length of ICU stay	20.6	69.6	67.2	42.5
Length of in-hospital stay	48.0	31.2	94.0	26.9

*COT* conventional oxygen therapy, *NIV* noninvasive ventilation, *HFOT* high-flow oxygen therapy, *ICU* intensive care unit

**Fig. 5:**
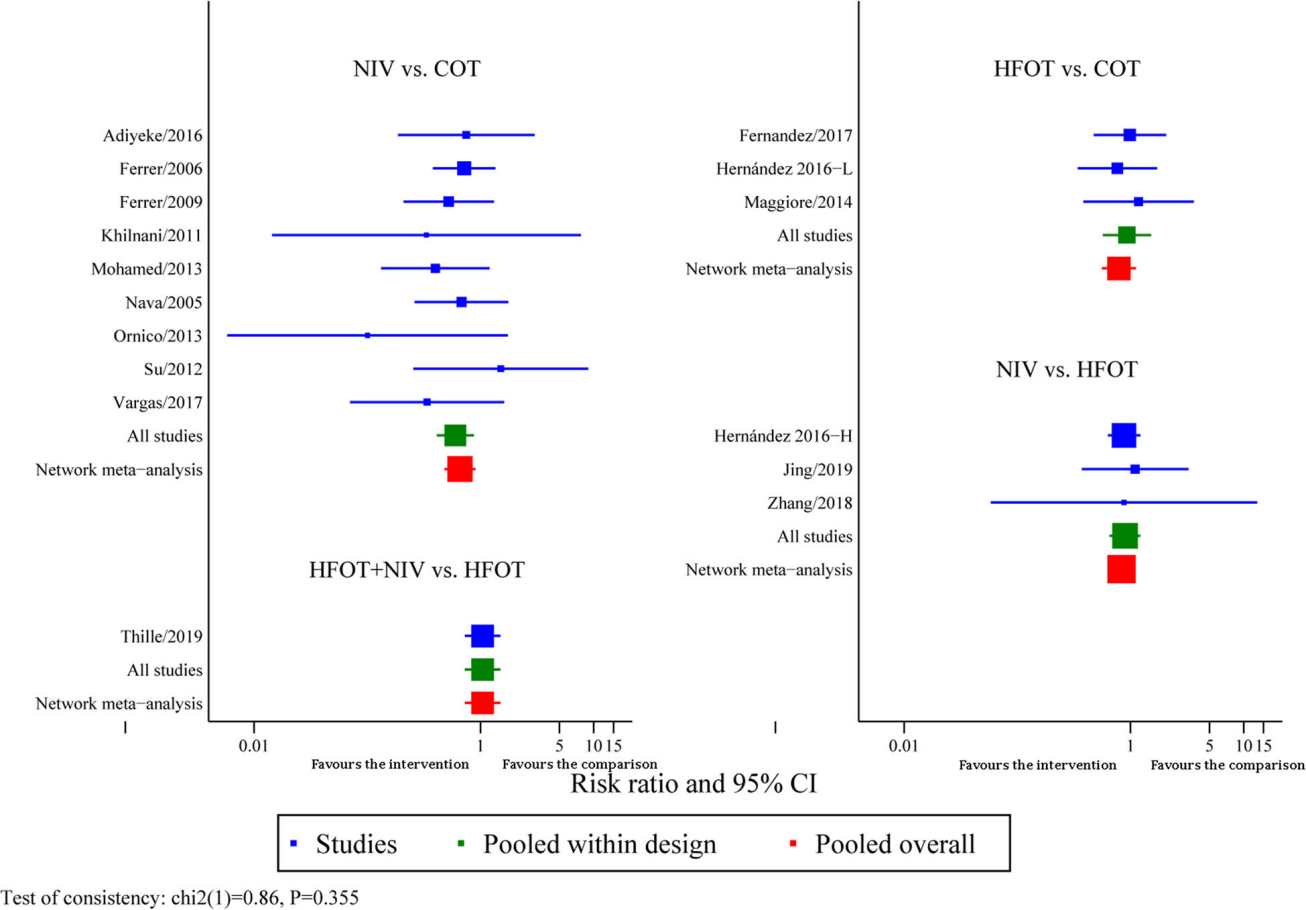
Forest plot of network meta-analysis for short-term mortality. NIV noninvasive ventilation, HFOT high-flow oxygen therapy, COT conventional oxygen therapy, CI confidence interval

Sixteen RCTs [23, 26–28, 37–46, 48, 49] enrolling 3281 patients reported short-term mortality. We found no heterogeneity across the included trials, and no significant inconsistency existed in this network (Table 1, Fig. S2 in Additional file 1). NMA estimates provided moderate to low-quality evidence and indicated that compared to COT, NIV decreased the risk of short-term death (RR 0.66, 95% CI 0.48 to 0.91) (Fig. 5, Table 1). HFOT had no beneficial effect on the short-term mortality compared with COT (RR 0.79, 95% CI 0.56 to 1.12). We found no difference in short-term mortality among NIV, HFOT, and HFOT+NIV (Table 2). NIV ranked first among the four respiratory support methods (Table 3).

**Fig. 6:**
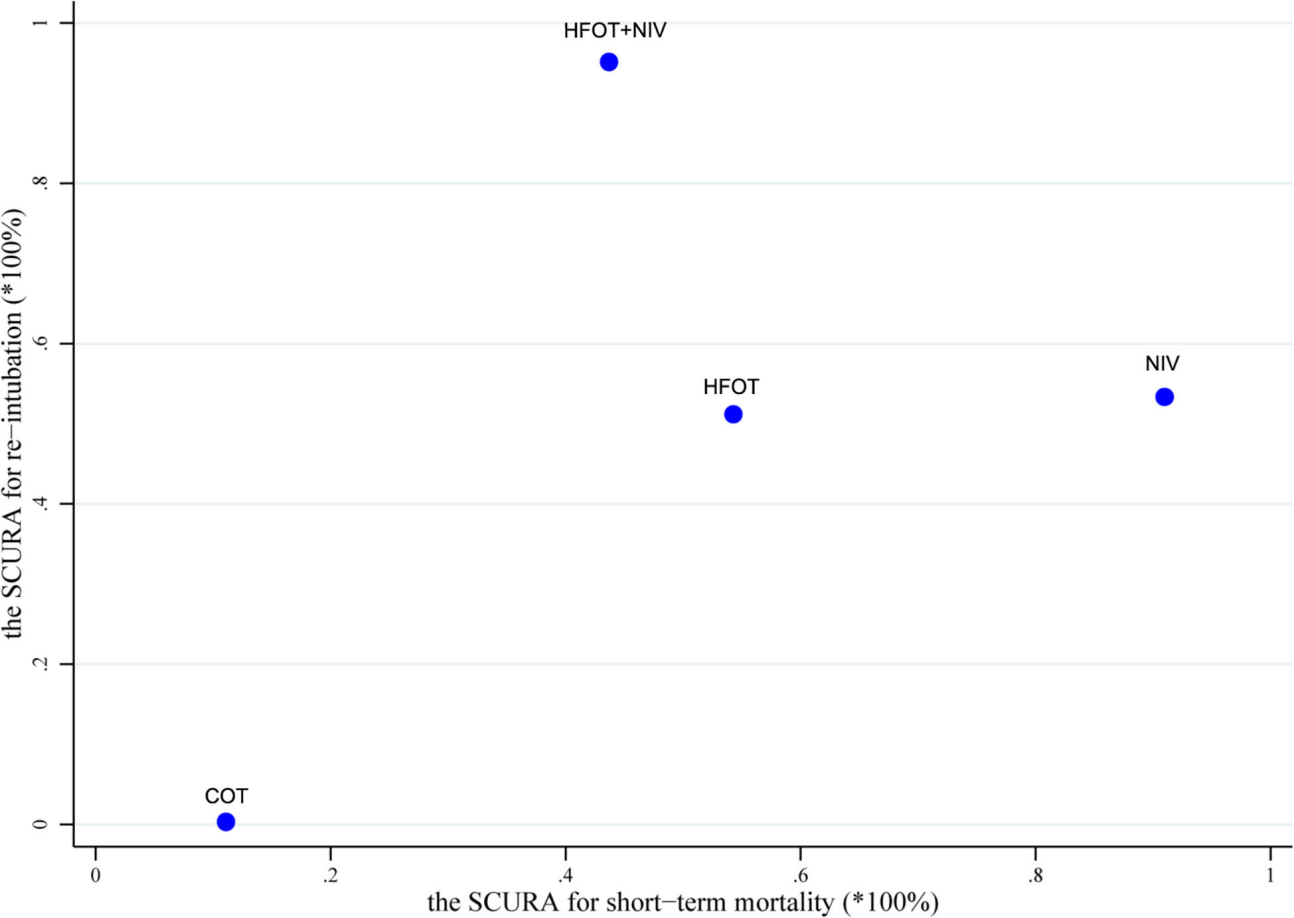
Clustered ranking plot based on cluster analysis of SUCRA values for the two primary outcomes. Treatment lying in the upper right corner is more effective in preventing re-intubation and short-term mortality than the other treatments. SCURA surface under the cumulative ranking curve, NIV noninvasive ventilation, HFOT high-flow oxygen therapy, COT conventional oxygen therapy

Although HFOT+NIV ranked highest for prevention of re-intubation, indirect evidence suggested that compared to NIV, HFOT+NIV likely increased short-term mortality (Table 2). In summary, NIV is probably the most effective method for comprehensively preventing re-intubation and short-term death (Fig. 6).

The network geometry and weight contribution matrix for the primary outcomes are shown in Fig. S3-S6 (see Additional file 1).

### Sensitivity analysis

Two sensitivity analyses were performed for the primary outcomes by exclusively using 14 trials that enrolled patients with a high risk of extubation failure or 11 trials that enrolled patients with PaCO_2_ < 45 mmHg at the end of SBT. The results suggested that the comparative effectiveness of various methods remained similar, and the SUCRA rankings were comparable to those of the primary analysis (Additional file 1: Table S4 and S5, Fig. S7-S10).

### Analysis of secondary outcomes

Eleven trials [23, 26–28, 37, 38, 43–46, 48] reported post-extubation respiratory failure. Heterogeneity was statistically significant across trials in the comparison of NIV and COT (Table 1). The consistent assumption in this network was acceptable (Fig. S11 in Additional file 1). The network estimates were ranked as moderate to low quality. Both NIV and HFOT were superior to COT in preventing post-extubation respiratory failure (Table 1, Fig. S12 in Additional file 1). Although HFOT+NIV had the highest SCURA value, its 95% CI was wide and contained the null effect when compared with NIV or HFOT (Table 2). Therefore, the treatment ranking should be interpreted with caution.

Fifteen trials [23, 26–28, 37–42, 44–46, 48, 49] reported the length of ICU stay. Substantial heterogeneity was noted across trials within the comparison of NIV and COT (Table 1). A problematic incoherence was found by visually inspecting the direct and indirect estimates despite no statistical significance (Additional file 1: Fig. S13 and S14). No evidence revealed the superiority of one particular respiratory support method because all the confidence intervals were very wide and included the null value (Table 1). Thus, the rank order should be interpreted cautiously.

Length of in-hospital stay was reported in 8 trials [23, 26–28, 38, 39, 41, 45]. We found a suspicious inconsistency in this network through visual inspection of the direct and indirect estimates (Additional file 1: Fig. S15 and S16). The network estimates provided low- to very low-quality evidence of no difference among COT, NIV, HFOT, and HFOT+NIV in terms of length of in-hospital stay (Tables 1 and 2). However, HFOT might reduce the length of in-hospital stay compared with COT (Table 1). HFOT ranked best among the four respiratory support methods (Table 3).

Only 3 RCTs reported the comfort score, of which 2 compared HFOT with COT [46, 47], and 1 compared HFOT with NIV [48]. Therefore, we did not perform an NMA for this outcome. According to the results from pairwise meta-analysis (Table 1), the comfort score of HFOT was lower than that of COT or NIV. The network geometry and weight contribution matrix for each secondary outcome are available in the supplementary material (Additional file 1: Fig. S17-S22).

## Discussion

This NMA of 17 RCTs comprising 3341 participants evaluated the relative effectiveness of four preventive respiratory support methods in critically ill medical patients. The findings suggested the superiority of NIV over COT in terms of re-intubation, short-term mortality, and post-extubation respiratory failure. Compared to COT, HFOT had beneficial effects on the re-intubation rate and post-extubation respiratory failure but not short-term mortality. There were similar treatment effects on the primary and secondary outcomes among NIV, HFOT, and HFOT+NIV. Although HFOT+NIV ranked best for reducing the risk of re-intubation, it exhibited the potential to increase short-term mortality compared with NIV. Therefore, to comprehensively prevent re-intubation and short-term death, prophylactic use of NIV after scheduled extubation is probably the most effective respiratory support method in critically ill medical patients, especially those with a high risk of extubation failure.

Respiratory support has been widely applied to prevent post-extubation respiratory failure, treat respiratory failure that developed after extubation, or facilitate early weaning from IMV in patients who have failed SBT [12]. Currently, routine use of COT remains the mainstay of preventive respiratory support in post-extubated patients. Since the low-flow oxygen delivered by COT is insufficient to generate positive airway pressure, COT may not guarantee adequate gas exchange to meet the demand of critically ill patients, especially those who were intubated for medical diseases, such as heart failure and COPD. Several RCTs have proven that preventive use of NIV or HFOT after planned extubation was an effective alternative approach [26, 37, 38, 41, 42, 44–46]. However, the most recent pairwise meta-analysis by Maitra et al. [19] concluded that NIV was not superior to COT in terms of prevention of re-intubation or death. Interestingly, in addition to trials that used NIV as a preventive strategy after planned extubation, the meta-analysis by Maitra et al. [19] also included trials in which NIV was applied as a facilitative or therapeutic strategy and trials in which patients received unplanned extubation. Moreover, at least two studies [37, 40] were missed in their meta-analysis [19]. Thus, substantial heterogeneity was identified across the included trials in their study [19], and their evidence had a low quality. Regarding the comparison of HFOT and COT, the two latest meta-analyses [20, 22] drew contradictory conclusions. The meta-analysis by Xu et al. [22] found a beneficial effect of HFOT on re-intubation. However, the meta-analysis by Zhu et al. [20] revealed no effects on re-intubation or mortality with the use of HFOT. Unfortunately, the two meta-analyses included a heterogeneous population that comprised post-surgical patients and critically ill medical patients. Moreover, the study by Zhu et al. [20] pooled results from RCTs and crossover studies. These factors might contribute to the above conflicting results. In contrast, a relatively homogeneous population of critically ill medical patients who received preventive respiratory support after planned extubation was recruited in our NMA. Network estimates suggested the benefits of NIV on the re-intubation rate and short-term mortality and the benefit of HFOT on the re-intubation rate compared with COT. These findings raised the question of why the benefits of HFOT on re-intubation could not be translated into survival benefits, but NIV could. It might be explained by the following: first, NIV could provide a higher positive airway pressure than HFOT. The high positive airway pressure delivered by NIV increases the intrathoracic pressure, which is analogous to IMV; reduces the left ventricular preload and afterload; and improves the cardiac performance [12]; these features might translate into a better prognosis in medical patients with cardiac failure. In addition, COPD is another one primary disease leading to ICU admission in most of the included trials in our study. Due to the generation of higher positive airway pressure, NIV may be more effective than HFOT in facilitating decarboxylation in COPD patients. This may be another one reason for interpreting the survival benefits of NIV. Finally, HFOT is more comfortable compared with COT or NIV [46–48]. For patients treated with noninvasive respiratory support that would fail, apparent improvement in patients comfort could mask deterioration to some extent [27] because prolonged noninvasive respiratory support resulting from comfort and tolerance could improve oxygenation ostensibly and thus might disguise signs of respiratory distress for an extended period [45] and ultimately lead to delayed re-intubation and increased mortality. This view is supported by the results of the study by Kang et al. [50]. This study suggested that the failure of HFOT might delay intubation and increase mortality.

There is limited information about the comparison of HFOT and NIV in post-extubated patients. Theoretically, preventive HFOT might be noninferior to NIV in post-extubated patients because HFOT had some distinctive advantages over NIV, including increased comfort [46–48, 51], easier clearance of secretions [52], and reduced risk of adverse effects [51, 53]. A multicenter RCT [27] found similar effects on the re-intubation rate and ICU mortality between HFOT and NIV. Our NMA also further confirmed the similar effectiveness of NIV and HFOT on the re-intubation rate and short-term mortality, which was consistent with the results of previous meta-analyses [21, 22]. Combinational use of HFOT and NIV seems to be a promising method in post-extubated patients because the addition of HFOT to NIV could, at least theoretically, further improve gas exchange and decrease the work of breathing. The HIGH-WEAN study [23] indicated that re-intubation rate and post-extubation respiratory failure were reduced with HFOT+NIV compared with HFOT alone. Our NMA also suggested that HFOT+NIV ranked first for the prevention of re-intubation rate and post-extubation respiratory failure. However, only one study has compared HFOT+NIV with other methods to date, and its direct estimate suggested that the 95% CI contained the null effect. One should thus be cautious in the interpretation of these findings.

The risk of extubation failure and PaCO_2_ level at the end of SBT might be the effect modifiers in this NMA. Therefore, we conducted sensitivity analyses for the primary outcomes. Our evidence was in favor of the preventive use of NIV after planned extubation in high-risk critically ill medical patients, which was consistent with the conditional recommendation from the European Respiratory Society/American Thoracic Society clinical practice guidelines [34]. Sensitivity analysis also indicated that NIV remains the most effective method in nonhypercapnic critically ill medical patients. However, an observational study by Gong et al. [54] found a conflicting result that prophylactic NIV could not reduce re-intubation or hospital mortality in COPD patients with PaCO_2_ < 45 mmHg. It is noteworthy that in our NMA, most trials that enrolled nonhypercapnic patients had a high risk of extubation failure. In addition, high-risk factors were not limited to COPD; they also comprised other factors, which might be one of the causes of the above conflicting results.

Several strengths in this NMA should be mentioned. First, to our knowledge, this is the first NMA to evaluate the comparative efficacies of various respiratory support methods in post-extubated critically ill patients. NMA allows the comparison of multiple treatments simultaneously in a single analysis and improves the precision by combining direct and indirect estimates. Second, a more homogeneous population was enrolled in this NMA. To improve the transitivity across comparisons and reduce the heterogeneity across included trials, we set strict inclusion criteria that only critically ill medical patients who were treated with preventive respiratory support after planned extubation could be included. Third, we performed sensitivity analyses to eliminate the influence of two potential effect modifiers on the NMA results and confirmed the robustness of the NMA results. Other strengths included a comprehensive literature search and application of GRADE methodology to assess the quality of evidence.

This NMA had some limitations. First, we did not construct a comparison-adjusted funnel plot to assess the presence of small-study effects due to limited studies in each direct comparison. Therefore, the possible overestimation of effect size in studies with a small sample size should be considered when interpreting the results. Second, although it is difficult to identify the effect modifiers in an NMA, we performed two sensitivity analyses to assess the robustness of the NMA results. However, there were also other differences among the included studies that potentially influenced the NMA results, including sample size, duration of respiratory support, and primary disease lead to IMV. Unfortunately, we did not perform sensitivity analysis for these factors given the limited information in the included studies. Third, the adverse complications were not analyzed in this NMA because the definition of adverse complications was largely different among included trials. Thus, we had no insight into the safety of various respiratory support approaches. Fourth, the pooled results of this NMA might have a potential bias given the lack of blindness in all included trials. Finally, we redefined high risk of extubation failure according to the previously published studies given that no consistent definition of “high risk” is available to date. As a consequence, this limitation may impact the certainty of the sensitivity analysis results.

## Conclusion

In critically ill medical patients, especially those who are at high risk of extubation failure, preventive use of NIV after scheduled extubation is probably the most effective respiratory support method for comprehensively preventing re-intubation and short-term death. This network meta-analysis showed a promising result for HFOT+NIV to prevent re-intubation. However, sufficient evidence regarding head-to-head comparisons of HFOT+NIV and other methods is still lacking. More high-quality studies comparing HFOT+NIV to other modalities of respiratory support are needed in the future.

## Supplementary information

**Additional file 1.** Detailed search strategies and supplementary tables and figures

## Data Availability

All data generated or analyzed during this study are included in this published article (and its supplementary information files).

## Abbreviations

IMV: Invasive mechanical ventilation
ICU: Intensive care unit
COT: Conventional oxygen therapy
FiO_2_: Fraction of inspired oxygen
NIV: Noninvasive ventilation
HFOT: High-flow oxygen therapy
RCTs: Randomized controlled trials
NMA: Network meta-analysis
PRISMA: Preferred Reporting Items for Systematic Reviews and Meta-Analyses
SBT: Spontaneous breathing trial
PaCO_2_: Atrial partial pressure of carbon dioxide
COPD: Chronic obstructive pulmonary disease
RR: Risk ratio
MD: Mean difference
CI: Confidence interval
SUCRA: Surface under the cumulative ranking curve
