# Supplementary material for: Preventive use of respiratory support after scheduled extubation in critically ill medical patients—a network meta-analysis of randomized controlled trials
Source: Crit Care. 2020 Jun 22;24:370. doi: 10.1186/s13054-020-03090-3 (PMC7306939; doi:10.1186/s13054-020-03090-3)

**SUPPLEMENTARY MATERIALS**

Table of contents

[Detailed search strategies for each database 2](#_Toc41501467)

[Table S1. Reasons for exclusion of irrelevant studies 6](#_Toc41501468)

[Table S2. Detailed characteristics of each included studies 12](#_Toc41501469)

[Table S3. Detailed outcomes from the included studies 21](#_Toc41501470)

[Table S4. Sensitivity analyses for the primary outcomes 24](#_Toc41501471)

[Table S5. Sensitivity analyses for the SCURA statistics 25](#_Toc41501472)

[Figure S1. Inconsistency analysis for the re-intubation rate 26](#_Toc41501473)

[Figure S2. Inconsistency analysis for the short-term mortality 27](#_Toc41501474)

[Figure S3. Network geometry for the re-intubation rate 28](#_Toc41501475)

[Figure S4. Weight contribution matrix for the re-intubation rate 29](#_Toc41501476)

[Figure S5. Network geometry for the short-term mortality 30](#_Toc41501477)

[Figure S6. Weight contribution matrix for the short-term mortality 31](#_Toc41501478)

[Figure S7. Forest plot of sensitivity analysis exclusively including trials with high risk of extubation failure for the re-intubation rate 32](#_Toc41501479)

[Figure S8. Forest plot of sensitivity analysis exclusively including trials with high risk of extubation failure for the short-term mortality 33](#_Toc41501480)

[Figure S9. Forest plot of sensitivity analysis exclusively including trials with PaCO_2_ < 45 mmHg at the end of SBT for the re-intubation rate 34](#_Toc41501481)

[Figure S10. Forest plot of sensitivity analysis exclusively including trials with PaCO_2_ < 45 mmHg at the end of SBT for the short-term mortality 35](#_Toc41501482)

[Figure S11. Inconsistency analysis for the post-extubation respiratory failure 36](#_Toc41501483)

[Figure S12. Forest plot of network meta-analysis for the post-extubation respiratory failure 37](#_Toc41501484)

[Figure S13. Inconsistency analysis for the length of ICU stay 38](#_Toc41501485)

[Figure S14. Forest plot of network meta-analysis for the length of ICU stay 39](#_Toc41501486)

[Figure S15. Inconsistency analysis for the length of in-hospital stay 40](#_Toc41501487)

[Figure S16. Forest plot of network meta-analysis for the length of in-hospital stay 41](#_Toc41501488)

[Figure S17. Network geometry for the post-extubation respiratory failure 42](#_Toc41501489)

[Figure S18. Weight contribution matrix for the post-extubation respiratory failure 43](#_Toc41501490)

[Figure S19. Network geometry for the length of ICU stay 44](#_Toc41501491)

[Figure S20. Weight contribution matrix for the length of ICU stay 45](#_Toc41501492)

[Figure S21. Network geometry for the length of in-hospital stay 46](#_Toc41501493)

[Figure S22. Weight contribution matrix for the length of in-hospital stay 47](#_Toc41501494)

# Detailed search strategies for each database

**PubMed: 147 records**

1. ((((Cannula[MeSH Terms]) OR Nasal Cannula) OR Cannula, Nasal) OR Nasal Cannulae) OR Cannulae, Nasal (15484 records)
2. (high-flow) OR high flow (161322 records)
3. (#1 AND #2) (1344 records)
4. ((HFNC) OR HHFNC) OR HHFN (422 records)
5. ((((((oxygen inhalation therapy[MeSH Terms]) OR Inhalation Therapy, Oxygen) OR Inhalation Therapies, Oxygen) OR Oxygen Inhalation Therapies) OR Therapies, Oxygen Inhalation) OR Therapy, Oxygen Inhalation) OR oxygen therapy (111460 records)
6. #2 AND #5 (2836 records)
7. #3 OR #4 OR #6 (3532 records)
8. ((((((((((non-invasive ventilation[MeSH Terms]) OR Noninvasive Ventilation*) OR Ventilation*, Noninvasive) OR Non-Invasive Ventilation*) OR Ventilation*, Non-Invasive) OR Ventilation*, Non-Invasive) OR Non Invasive Ventilation*) OR Ventilation*, Non Invasive) OR noninvasive positive pressure ventilation) OR non-invasive positive pressure ventilation) OR NIPPV (8187 records)
9. (((conventional oxygen therapy) OR COT) OR standard oxygen therapy) OR SOT (12712 records)
10. ((venturi mask) OR face mask) OR bag valve mask (4987 records)
11. #5 OR #9 OR #10 (120742 records)
12. #7 OR #8 OR #11 (127837 records)
13. ((((((((((((((Airway Extubation[MeSH Terms]) OR Airway Extubation*) OR Extubation*, Airway) OR Tracheal Extubation*) OR Extubation*, Tracheal) OR Extubation*, Intratracheal) OR Intratracheal Extubation*) OR Endotracheal Extubation*) OR Extubation*, Endotracheal) OR postextubation) OR post-extubation) OR after extubation*) OR following extubation*) OR extubated) OR extubation* (13153 records)
14. ((((((Critical Care[MeSH Terms]) OR intensive care[MeSH Terms]) OR Critical Illness[MeSH Terms]) OR Intensive Care Units[MeSH Terms])) OR ((((((((Critical Care) OR intensive care) OR Critical* illness) OR Intensive Care Unit*) OR ICU) OR ICUs) OR intensive illness) OR critically ill)) (263052 records)
15. #12 AND #13 AND #14 Filters: Clinical Trial; Humans (147 records)

**Embase: 2195 records**

1. ('cannula'/exp OR 'cannula' OR 'nasal cannula'/exp OR 'nasal cannula' OR 'cannula, nasal' OR 'nasal cannulae' OR 'cannulae, nasal') AND ('high-flow' OR 'high flow') (2106 )
2. ('oxygen inhalation therapy'/exp OR 'oxygen inhalation therapy' OR 'inhalation therapy, oxygen' OR 'inhalation therapies, oxygen' OR 'oxygen inhalation therapies' OR 'therapies, oxygen inhalation' OR 'therapy, oxygen inhalation' OR 'oxygen therapy'/exp OR 'oxygen therapy') AND ('high-flow' OR 'high flow') (1355 records)
3. 'hfnc' OR 'hhfnc' OR 'hhfn' (946 records)
4. #1 OR #2 OR #3 (2719 records)
5. 'conventional oxygen therapy' OR 'cot' OR 'standard oxygen therapy' OR 'sot' OR 'venturi mask'/exp OR 'venturi mask' OR 'face mask'/exp OR 'face mask' OR 'bag valve mask'/exp OR 'bag valve mask' OR 'oxygen inhalation therapy':au OR 'inhalation therapy, oxygen':au OR 'inhalation therapies, oxygen':au OR 'oxygen inhalation therapies':au OR 'therapies, oxygen inhalation':au OR 'therapy, oxygen inhalation':au OR 'oxygen therapy' (17106 records)
6. 'non-invasive ventilation'/exp OR 'non-invasive ventilation' OR 'noninvasive ventilation*' OR 'ventilation*, noninvasive' OR 'non-invasive ventilation*' OR 'ventilation*, non-invasive' OR 'non invasive ventilation*' OR 'ventilation*, non invasive' OR 'noninvasive positive pressure ventilation'/exp OR 'noninvasive positive pressure ventilation' OR 'non-invasive positive pressure ventilation' OR 'nippv' (21307 records)
7. #4 OR #5 OR #6 (38683 records)
8. 'airway extubation'/exp OR 'airway extubation' OR 'airway extubation*' OR 'extubation*, airway' OR 'tracheal extubation*' OR 'extubation*, tracheal' OR 'extubation*, intratracheal' OR 'intratracheal extubation*' OR 'endotracheal extubation*' OR 'extubation*, endotracheal' OR 'postextubation' OR 'post-extubation' OR 'after extubation*' OR 'following extubation*' OR 'extubated' OR 'extubation*' (28837 records)
9. 'critical care'/exp OR 'critical care' OR 'intensive care'/exp OR 'intensive care' OR 'critical illness'/exp OR 'critical illness' OR 'critical* illness' OR 'intensive care unit*' OR 'icu' OR 'icus' OR 'intensive illness' OR 'critically ill'/exp OR 'critically ill' (1169421 records)
10. #7 AND #8 AND #9 AND 'human'/de (2195 records)

**Cochrane Central Register of Controlled Trials: 526 records**

1. MeSH descriptor: [Cannula] explode all trees (55 records)
2. (Nasal Cannula) OR (Cannula*, Nasal) OR (Nasal Cannula*) (1294 records)
3. #1 OR #2 (1302 records)
4. (high-flow) OR (high flow) (16655 records)
5. #3 AND #4 (811 records)
6. (HFNC) OR (HHFNC) OR (HHFN) (357 records)
7. MeSH descriptor: [Oxygen Inhalation Therapy] explode all trees (1464 records)
8. (Inhalation Therapy, Oxygen) OR (Inhalation Therapies, Oxygen) OR (Oxygen Inhalation Therapies) OR (Therapies, Oxygen Inhalation) OR ((Therapy, Oxygen Inhalation) OR (oxygen therapy)) (19626 records)
9. #7 OR #8 (18294 records)
10. #4 AND #9 (2011 records)
11. (Noninvasive Ventilation*) OR (Ventilation*, Noninvasive) OR (Non-Invasive Ventilation*) OR (Ventilation*, Non-Invasive) OR (Ventilation*, Non-Invasive) OR (Non Invasive Ventilation*) OR (Ventilation*, Non Invasive) OR (noninvasive positive pressure ventilation) OR (non-invasive positive pressure ventilation) OR (NIPPV) (3578 records)
12. MeSH descriptor: [Noninvasive Ventilation] explode all trees (205 records)
13. #11 OR #12 (3578 records)
14. (conventional oxygen therapy) OR (COT) OR (standard oxygen therapy) OR (SOT) OR (venturi mask) OR (face mask) OR (bag valve mask) (7033 records)
15. #9 OR #14 (20565 records)
16. #5 OR #6 OR #10 OR #13 OR #15 (23251 records)
17. MeSH descriptor: [Airway Extubation] explode all trees (203 records)
18. (Airway Extubation*) OR (Extubation*, Airway) OR (Tracheal Extubation*) OR (Extubation*, Tracheal) OR (Extubation*, Intratracheal) OR (Intratracheal Extubation*) OR (Endotracheal Extubation*) OR (Extubation*, Endotracheal) OR (postextubation) OR (post-extubation) OR (after extubation*) OR (following extubation*) OR (extubated) OR (extubation*) (6376 records)
19. #17 OR #18 (6376 records)
20. MeSH descriptor: [Critical Illness] explode all trees (1972 records)
21. MeSH descriptor: [Intensive Care Units] explode all trees (3439 records)
22. MeSH descriptor: [Critical Care] explode all trees (1998 records)
23. (Critical Care) OR (intensive care) OR (Critical Illness) OR (Critical* illness) OR (Intensive Care Unit*) OR (ICU) OR (ICUs) OR (intensive illness) OR (critically ill) (61273 records)
24. #20 OR #21 OR #22 OR #23 (61487 records)
25. #16 AND #19 AND #24 Limits: in Trials (526 records)

**Web of Science: 598 records**

1. TS= (Nasal Cannula) OR TS= (Cannula, Nasal) OR TS= (Nasal Cannulae) OR TS= (Cannulae, Nasal) (2259 records)
2. TS=(high-flow) OR TS=(high flow) (657279 records)
3. #1 AND #2 (1374 records)
4. TS=(HFNC) OR TS=(HFNC) OR TS=(HHFN) (393 records)
5. TS=(oxygen inhalation therapy) OR TS=(Inhalation Therapy, Oxygen) OR TS=(Inhalation Therapies, Oxygen) OR TS=(Oxygen Inhalation Therapies) OR TS=(Therapies, Oxygen Inhalation) OR TS=(Therapy, Oxygen Inhalation) OR TS=(oxygen therapy) (51695 records)
6. #2 AND #5 (2503 records)
7. #3 OR #4 OR #6 (3190 records)
8. TS=(conventional oxygen therapy) OR TS=(COT) OR TS=(standard oxygen therapy) OR TS=(SOT) OR TS=(venturi mask) OR TS=(face mask) OR TS=(bag valve mask) OR TS=(oxygen inhalation therapy) OR TS=(Inhalation Therapy, Oxygen) OR TS=(Inhalation Therapies, Oxygen) OR TS=(Oxygen Inhalation Therapies) OR TS=(Therapies, Oxygen Inhalation) OR TS=(Therapy, Oxygen Inhalation) OR TS=(oxygen therapy) OR TS=(non-invasive ventilation) OR TS=(Noninvasive Ventilation*) OR TS=(Ventilation*, Noninvasive) OR TS=(Non-Invasive Ventilation*) OR TS=(Ventilation*, Non-Invasive) OR TS=(Ventilation*, Non-Invasive) OR TS=(Non Invasive Ventilation*) OR TS=(Ventilation*, Non Invasive) OR TS=(noninvasive positive pressure ventilation) OR TS=(non-invasive positive pressure ventilation) OR TS=(NIPPV) (83729 records)
9. #7 OR #8 (84235 records)
10. TS=(Airway Extubation) OR TS=(Airway Extubation*) OR TS=(Extubation*, Airway) OR TS=(Tracheal Extubation*) OR TS=(Extubation*, Tracheal) OR TS=(Extubation*, Intratracheal) OR TS=(Intratracheal Extubation*) OR TS=(Endotracheal Extubation*) OR TS=(Extubation*, Endotracheal) OR TS=(postextubation) OR TS=(post-extubation) OR TS=(after extubation*) OR TS=(following extubation*) OR TS=(extubated) OR TS=(extubation*) (11453 records)
11. TS=(Critical Care) OR TS=(intensive care) OR TS=(Critical Illness) OR TS=(Intensive Care Unit*) OR TS=(Critical* illness) OR TS=(ICU) OR TS=(ICUs) OR TS=(intensive illness) OR TS=(critically ill) (278961 records)
12. #9 AND #10 AND #11 (598 records)

# Table S1. Reasons for exclusion of irrelevant studies

| **Reasons for exclusion of irrelevant studies** | **Referenced studies** | |
| --- | --- | --- |
| Conducted in post-surgical patients, not in critically ill medical patients (17 studies) | Al Jaaly/2013 [1] | Brainard/2017 [2] |
|  | Chen/2017 [3] | Corley/2015 [4] |
|  | de Araújo-Filho/2017 [5] | Ferrando/2019 [6] |
|  | Futier/2016 [7] | Gust/1996 [8] |
|  | Jaber/2016 [9] | Lopes/2008 [10] |
|  | Parke/2013 [11] | Stéphan/2015 [12] |
|  | Yang/2016 [13] | Yang/2017 [14] |
|  | Yu/2017 [15] | Zhu/2013 [16] |
|  | Zochios/2018 [17] |  |
| Abstract without the full-text (9 studies) | Antonicelli/2011 [18] | Arman/2017 [19] |
|  | Canovas/2014 [20] | Cracchiolo/2017 [21] |
|  | Gaspari/2019 [22] | Golukhova/2011 [23] |
|  | Naumov/2011 [24] | Perbet/2014 [25] |
|  | Youssef/2018 [26] |  |
| Studies were not randomized controlled trial (6 studies) | Boeken/2010 [27] | Duan/2016 [28] |
|  | Eremenko/2019 [29] | Ezingeard/2006 [30] |
|  | Gaspari/2020 [31] | Kindgen-Milles/2000 [32] |
| Studies were not conducted after extubation (1 study) | Martin/2000 [33] | |
| Subjects were not critically ill medical patients admitted to the ICU (4 studies) | Pennisi/2019 [34] | Wong/2011 [35] |
|  | Zoremba/2011 [36] | Guimarães/2016 [37] |
| Subjects undergone a unplanned extubation (2 studies) | Hsu/2012 [38]; Jiang [39] | |
| Ventilatory support (HFOT or NIV) was used as therapeutic strategy (5 studies) | Esteban/2004 [40] | Keenen/2002 [41] |
|  | Hou/2019 [42] | Zhu/2017 [43] |
|  | Matsuda/2020[44] |  |
|  | The above four studies were conducted in patients who had developed to respiratory failure after extubation, and the ventilatory support, HFOT or NIV, was used after extubation as therapeutic strategy which was defined by Maggiore et al. [45] as use of HFOT or NIV after extubation in patients with post-extubation acute respiratory failure. | |
| Ventilatory support (NIV) was used as facilitative strategy (1 study) | Girault/2011 [46] | |
|  | This study was conducted in chronic hypercapnic respiratory failure patients who experienced an early extubation despite intolerance of spontaneous breathing trial, and the ventilatory support, NIV, was used after extubation as facilitative strategy which was defined by Maggiore et al. [45] as use of NIV after early extubation in selected patients who have failed the spontaneous breathing trial. | |

**Reference**

1. Al Jaaly E, Fiorentino F, Reeves BC, Ind PW, Angelini GD, Kemp S, et al. Effect of adding postoperative noninvasive ventilation to usual care to prevent pulmonary complications in patients undergoing coronary artery bypass grafting: a randomized controlled trial. J Thorac Cardiovasc Surg. 2013;146(4):912-8.
2. Brainard J, Scott BK, Sullivan BL, Fernandez-Bustamante A, Piccoli JR, Gebbink MG, et al. Heated humidified high-flow nasal cannula oxygen after thoracic surgery - A randomized prospective clinical pilot trial. J Crit Care. 2017;40:225-228.
3. Chen XF, Ye JL. Efficacy and safety of non-invasive positive pressure ventilation in the care of dyspnea after cardiac surgery. Zhongguo Wei Zhong Bing Ji Jiu Yi Xue. 2007;19(9):542-5. [Article in Chinese]
4. Corley A, Bull T, Spooner AJ, Barnett AG, Fraser JF. Direct extubation onto high-flow nasal cannulae post-cardiac surgery versus standard treatment in patients with a BMI ≥30: a randomised controlled trial. Intensive Care Med. 2015;41(5):887-94.
5. de Araújo-Filho AA, de Cerqueira-Neto ML, de Assis Pereira Cacau L, Oliveira GU, Cerqueira TCF, de Santana-Filho VJ. Effect of prophylactic non-invasive mechanical ventilation on functional capacity after heart valve replacement: a clinical trial. Clinics (Sao Paulo). 2017;72(10):618-623.
6. Ferrando C, Puig J, Serralta F, Carrizo J, Pozo N, Arocas B, et al. High-flow nasal cannula oxygenation reduces postoperative hypoxemia in morbidly obese patients: a randomized controlled trial. Minerva Anestesiol. 2019;85(10):1062-1070.
7. Futier E, Paugam-Burtz C, Godet T, Khoy-Ear L, Rozencwajg S, Delay JM, et al. Effect of early postextubation high-flow nasal cannula vs conventional oxygen therapy on hypoxaemia in patients after major abdominal surgery: a French multicentre randomised controlled trial (OPERA). Intensive Care Med. 2016;42(12):1888-1898.
8. Gust R, Gottschalk A, Schmidt H, Böttiger BW, Böhrer H, Martin E. Effects of continuous (CPAP) and bi-level positive airway pressure (BiPAP) on extravascular lung water after extubation of the trachea in patients following coronary artery bypass grafting. Intensive Care Med. 1996;22(12):1345-50.
9. Jaber S, Lescot T, Futier E, Paugam-Burtz C, Seguin P, Ferrandiere M, et al. Effect of Noninvasive Ventilation on Tracheal Reintubation Among Patients With Hypoxemic Respiratory Failure Following Abdominal Surgery: A Randomized Clinical Trial. JAMA. 2016;315(13):1345-53.
10. Lopes CR, Brandão CM, Nozawa E, Auler JO Jr. Benefits of non-invasive ventilation after extubation in the postoperative period of heart surgery. Rev Bras Cir Cardiovasc. 2008;23(3):344-50.
11. Parke R, McGuinness S, Dixon R, Jull A. Open-label, phase II study of routine high-flow nasal oxygen therapy in cardiac surgical patients. Br J Anaesth. 2013 ;111(6):925-31.
12. Stéphan F, Barrucand B, Petit P, Rézaiguia-Delclaux S, Médard A, Delannoy B, et al. High-Flow Nasal Oxygen vs Noninvasive Positive Airway Pressure in Hypoxemic Patients After Cardiothoracic Surgery: A Randomized Clinical Trial. JAMA. 2015;313(23):2331-9.
13. Yang Y, Liu N, Sun L, Zhou Y, Yang Y, Shang W, et al. Noninvasive Positive-Pressure Ventilation in Treatment of Hypoxemia After Extubation Following Type-A Aortic Dissection. J Cardiothorac Vasc Anesth. 2016;30(6):1539-1544.
14. Yang Y, Liu N, Sun LZ, Yang Y. Effects of prophylactic use of noninvasive positive pressure ventilation following stanford type A aortic dissection operation. Chin J Thorac Cardiovasc Surg. 2017, 33(2): 81-86. [Article in Chinese]
15. Yu Y, Qian X, Liu C, Zhu C. Effect of High-Flow Nasal Cannula versus Conventional Oxygen Therapy for Patients with Thoracoscopic Lobectomy after Extubation. Can Respir J. 2017; 2017:7894631.
16. Zhu GF, Wang DJ, Liu S, Jia M, Jia SJ. Efficacy and safety of noninvasive positive pressure ventilation in the treatment of acute respiratory failure after cardiac surgery. Chin Med J (Engl). 2013;126(23):4463-9.
17. Zochios V, Collier T, Blaudszun G, Butchart A, Earwaker M, Jones N, et al. The effect of high-flow nasal oxygen on hospital length of stay in cardiac surgical patients at high risk for respiratory complications: a randomised controlled trial. Anaesthesia. 2018;73(12):1478-1488.
18. Antonicelli F, Cataldo A, Festa R, Idone F, Moccaldo A, Antonelli M, et al. High-flow oxygen therapy through nasal cannulae versus low-flow oxygen therapy via Venturi mask after extubation in adult, critically ill patients. Crit Care. 2011; 15(Suppl 1): P165.
19. Arman PD , Varn MN, Povian S , Davis A , Uchakin P, Bhar A, et al. Effects Of Direct Extubation To High-Flow Nasal Cannula Compared To Standard Nasal Cannula In Patients In The Intensive Care Unit. AMERICAN JOURNAL OF RESPIRATORY AND CRITICAL CARE MEDICINE. 2017, 195: A1887.
20. Canovas J, Lopez A, Fernandez M, Capilla L, Botias S, Alcazar M, et al. Effectiveness of noninvasive ventilation in the prevention of post-extubation respiratory failure. Intensive Care Med. 2014,40(1):S73.
21. Cracchiolo A N, Palma D M, Tetamo R. High flow nasal cannula vs standard oxygen face mask during physiotherapy in brain injury patients: A feasible study. Intensive Care Med Exp. 2017,5(2): L619044277.
22. Gaspari R, Spinazzola G, Ferrone G, Soave PM, Pintaudi G, Avolio AW, et al. Use of high-flow nasal cannula vs standard oxygen therapy via venturi mask in liver transplantation after extubation to prevent the hypoxemia: A matched-controlled study. Transplantation.2019,103(8):122.
23. Golukhova E, Medressova A, Luckashkin M, Lobacheva G, Shumkov K, Merzlyakov V. Non-invasive ventilation to prevent postoperative pulmonary complications after cardiac surgery. Interact Cardiovasc Thorac Surg. 2011,12: S42.
24. Naumov A, Khubulava G, Shikhverdiev N, Polushin Yu, Marchenko S, Povarenkov A, et al. Influence of non-invasive ventilation on hemodynamics and gas exchange in patients with heart failure after cardiac surgery. Interact Cardiovasc Thorac Surg. 2011,12: S108.
25. Perbet S, Gerst A, Chabanne R, Soummer A, Faure J S, Pascal J, et al. High-flow nasal oxygen cannula versus conventional oxygen therapy to prevent postextubation lung aeration loss: A multicentric randomized control lung ultrasound study. Intensive Care Med. 2014, 4(1): S128.
26. Youssef Z, Sara AS, Amra Z, Abdenasser SM. Prophylactic non invasive ventilation after extubation in severe brain injured patients. Annals of Intensive Care. 2018,8(1): 620836987.
27. Boeken U, Schurr P, Kurt M, Feindt P, Lichtenberg A. Early reintubation after cardiac operations: impact of nasal continuous positive airway pressure (nCPAP) and noninvasive positive pressure ventilation (NPPV). Thorac Cardiovasc Surg. 2010;58(7):398-402.
28. Duan J, Han X, Huang S, Bai L. Noninvasive ventilation for avoidance of reintubation in patients with various cough strength. Crit Care. 2016;20(1):316.
29. Eremenko AA, Polyakova PV, Vyzhigina MA. Influence of noninvasive respiratory support techniques on gas exchange in cardiac surgical patients suffering from post-operative respiratory failure. Obshchaya Reanimatologiya. 2019, 15(4): 21-31.
30. Ezingeard E, Diconne E, Guyomarc'h S, Venet C, Page D, Gery P, et al. Weaning from mechanical ventilation with pressure support in patients failing a T-tube trial of spontaneous breathing. Intensive Care Med. 2006;32(1):165-9.
31. Gaspari R, Spinazzola G, Ferrone G, Soave PM, Pintaudi G, Cutuli SL. High-Flow Nasal Cannula Versus Standard Oxygen Therapy After Extubation in Liver Transplantation: A Matched Controlled Study. Respir Care. 2020;65(1):21-28.
32. Kindgen-Milles D, Buhl R, Gabriel A, Böhner H, Müller E. Nasal continuous positive airway pressure: A method to avoid endotracheal reintubation in postoperative high-risk patients with severe nonhypercapnic oxygenation failure. Chest. 2000;117(4):1106-11.
33. Martin TJ, Hovis JD, Costantino JP, Bierman MI, Donahoe MP, Rogers RM, et al. A randomized, prospective evaluation of noninvasive ventilation for acute respiratory failure. Am J Respir Crit Care Med. 2000;161(3 Pt 1):807-13.
34. Pennisi MA, Bello G, Congedo MT, Montini L, Nachira D, Ferretti GM, et al. Early nasal high-flow versus Venturi mask oxygen therapy after lung resection: a randomized trial. Crit Care. 2019;23(1):68.
35. Wong DT, Adly E, Ip HY, Thapar S, Maxted GR, Chung FF. A comparison between the Boussignac™ continuous positive airway pressure mask and the venturi mask in terms of improvement in the PaO2/F(I)O2 ratio in morbidly obese patients undergoing bariatric surgery: a randomized controlled trial. Can J Anaesth. 2011;58(6):532-9.
36. Zoremba M, Kalmus G, Begemann D, Eberhart L, Zoremba N, Wulf H, et al. Short term non-invasive ventilation post-surgery improves arterial blood-gases in obese subjects compared to supplemental oxygen delivery - a randomized controlled trial. BMC Anesthesiol. 2011;11:10.
37. Guimarães J, Pinho D, Nunes CS, Cavaleiro CS, Machado HS. Effect of Boussignac continuous positive airway pressure ventilation on Pao2 and Pao2/Fio2 ratio immediately after extubation in morbidly obese patients undergoing bariatric surgery: a randomized controlled trial. J Clin Anesth. 2016;34:562-70.
38. Hsu, HW, Li, CH, Chen, MC, Chien, HT, Wang, CH, Wu, SH, et al. The Effectiveness of Immediate Non-invasive Positive Pressure Ventilation on Decreasing the Re-intubation Rate of Unplanned Extubation. Journal of Internal Medicine of Taiwan. 2012,23(5): 351-359.
39. Jiang JS, Kao SJ, Wang SN. Effect of early application of biphasic positive airway pressure on the outcome of extubation in ventilator weaning. Respirology. 1999; 4:161–165.
40. Esteban A, Frutos-Vivar F, Ferguson ND, Arabi Y, Apezteguía C, González M, et al. Noninvasive positive-pressure ventilation for respiratory failure after extubation. N Engl J Med. 2004;350(24):2452-60.
41. Keenan SP, Powers C, McCormack DG, Block G. Noninvasive positive-pressure ventilation for postextubation respiratory distress: a randomized controlled trial. JAMA. 2002;287(24):3238-44.
42. Hou Q, Zhang Z, Lei T, Gan M, Wu X, Yue W, et al. Clinical efficacy of high-flow nasal humidified oxygen therapy in patients with hypoxemia. PLoS One. 2019;14(6):e0216957.
43. Zhu Z, Liu Y, Wang Q, Wang S. Preliminary evaluation of sequential therapy by high flow nasal cannula oxygen therapy following endotracheal tube extubation in mechanically ventilated patients. Zhonghua Wei Zhong Bing Ji Jiu Yi Xue. 2017;29(9):778-782.
44. Matsuda W, Hagiwara A, Uemura T, Sato T, Kobayashi K, Sasaki R, et al. High-Flow Nasal Cannula May Not Reduce the Re-Intubation Rate after Extubation in Respiratory Failure Compared With a Large-Volume Nebulization-Based Humidifier. Respir Care. 2020. pii: respcare.07095. doi: 10.4187/respcare.07095. [Epub ahead of print]
45. Maggiore SM, Battilana M, Serano L, Petrini F. Ventilatory support after extubation in critically ill patients. Lancet Respir Med. 2018;6(12):948-962.
46. Girault C, Bubenheim M, Abroug F, Diehl JL, Elatrous S, Beuret P, et al. Noninvasive ventilation and weaning in patients with chronic hypercapnic respiratory failure: a randomized multicenter trial. Am J Respir Crit Care Med. 2011;184(6):672-9.

# Table S2. Detailed characteristics of each included studies

| First author /Publication year | Design (location) | No. of participants | | Population and risk of extubation failure | | The primary diagnosis leading to IMV | Experimental intervention  (EIG) | Control intervention  (CIG) | APACHE II/SAPS II score on admission | | | | | PaCO_2_ at the end of SBT (during SBT or at extubation) | | | | Study period | Outcomes of interest |
| --- | --- | --- | --- | --- | --- | --- | --- | --- | --- | --- | --- | --- | --- | --- | --- | --- | --- | --- | --- |
|  |  |  |  |  |  |  |  |  | EIG | CIG | | | | EIG | | | CIG |  |  |
| Adiyeke/2016 | Single-center (Turkey) | 50 | Acute RF patients treated with IMV > 48 hrs, fulfilled the criteria for SBT, and scheduled for extubation. | | Acute RF | | NIV (by full-face or oronasal-forehead or chin supported mask) was used with Bi-PAP mode after extubation | COT by venture face mask (VM) | 19.54 ±4.46 | | | 20.76±7.03 | | Unavailable | | Unavailable | | 48 hrs after extubation | Reintubation and RF within 48 hrs after extubation, ICU mortality, length of ICU stay |
|  |  |  | **High** risk of extubation failure | |  |  |  |  |  |  |  |  |  |  |  |  |  |  |  |
| Ferrer/2006 | Multicenter (2 ICUs in Spain) | 162 | Patients intubated for > 48 hrs and tolerated SBT and scheduled for extubation, and were at risk of RF after extubation. | | Exacerbation of chronic respiratory disorders, pneumonia, congestive heart failure, or other reasons | | NIV was continuously  delivered immediately after extubation using Bi-PAP mode | COT by VM | 22 ± 5 | | | 20 ± 6 | | 44 ± 10 | 42 ± 9 | | | 24 hrs after extubation | Reintubation and RF within 72 hrs after extubation, ICU and inhospital mortality, length of ICU and inhospital stay |
|  |  |  | **High** risk of extubation failure | |  |  |  |  |  |  |  |  |  |  |  |  |  |  |  |
| Ferrer/2009 | Multicenter (3 ICUs in Spain) | 106 | Patients with chronic respiratory disorders, intubated for > 48 hrs, tolerated SBT and scheduled for extubation, with PaCO2 > 45 mm Hg at the end of SBT. | | Exacerbation of chronic respiratory disorders, pneumonia, congestive heart failure,sepsis, or other reasons | | NIV (by face mask) was delivered continuously immediately after extubation using Bi-PAP mode to achieve SpO_2_ > 92% | COT by VM, to achieve arterial SpO_2_ > 92% | 20 ± 7 | | | 20 ± 7 | | 55 ± 6 | 53 ± 5 | | | 24 hrs after extubation | Reintubation and RF within 72 hrs after extubation, ICU and inhospital mortality, length of ICU and inhospital stay |
|  |  |  | **High** risk of extubation failure | |  |  |  |  |  |  |  |  |  |  |  |  |  |  |  |
| Khilnani/2011 | Single-center (India) | 40 | Patients with AECOPD and type-2 RF, received IMV for > 48 hrs, tolerated SBT and had planned extubation | | AECOPD with type-2 RF | | NIV with Bi-PAP mode by well-fitting full-facemask, at least 7 hrs a day, to keep an SpO_2_ ≥ 88% | COT by nasal prongs or  oronasal mask, to keep an SpO_2_ of ≥ 88% | Unavailable | | | Unavailable | | mean PaCO2 >50 mmHg | mean PaCO2 >50 mmHg | | | Study continued untill discomfort, deterioration in ventilatory parameters or consciousness state, or haemodynamic instability. | Reintubation, inhospital mortality, length of ICU and inhospital stay |
|  |  |  | **High** risk of extubation failure | |  |  |  |  |  |  |  |  |  |  |  |  |  |  |  |
| Mohamed/2013 | Single-center (Saudi Arabia) | 120 | Adult patients suffered RF and received IMV for > 48 hrs, and tolerated a weaning trial and scheduled for extubation | | COPD, pneumonia, sepsis, or other reasons | | NIV with a full facial mask using Bi-PAP mode, immediately after extubation, to maintain the arterial SpO_2_ > 90% | COT immediately after extubation through a facial mask with a flow of 5 L/min | 23.2±5.4 | | | 21.8± 6.1 | | 39.9 ±6.3 | 42.8 ±7.7 | | | Throughout their ICU stay | Reintubation in the ICU, ICU mortality, length of ICU stay |
|  |  |  | **High** risk of extubation failure | |  |  |  |  |  |  |  |  |  |  |  |  |  |  |  |
| Nava/2005 | Multicenter (3 ICUs in Italy) | 97 | ICU patients ventilated for > 48 hrs and were at high risk of postextubation RF, and tolerated SBT and scheduled for extubation | | COPD exacerbation, pneumonia, ARDS, or other reasons | | NIV, with full face mask or nasal mask, using either Bi-PAP ventilator or PSV +PEEP mode, to achieve SpO_2_ > 92%. | COT, with full face mask or nasal mask, was delivered to achieve SpO_2_ > 92% | 31.4±0.3^a^ | | | 32.5± 2.6^a^ | | 41.7± .26 | 39.4 ± 5.65 | | | 48 hrs after extubation | Reintubation in the ICU, ICU and hospital mortality  length of stay in the ICU and in hospital |
|  |  |  | **High** risk of extubation failure | |  |  |  |  |  |  |  |  |  |  |  |  |  |  |  |
| Ornico/2013 | Single-center (Brazil) | 38 | Patients with acute RF treated with IMV administered by orotracheal tube for > 72 hrs and were weaning from IMV by using the ICU weaning protocol | | Pneumonia, abdominal surgery, sepsis, or other reasons | | NIV was immediately administered with a Bi-PAP device in spontaneous mode after extubation for a continuous period of 24 hrs | COT immediately after extubation through a facial mask with a flow of 5 L/min. | 16.90±6.81 | | | 15.28±5.65 | | 34.56±.43 | 38.31± 4.74 | | | 24 hrs after extubation | Reintubation within 48 hrs after extubation, hospital mortality  length of ICU stay |
|  |  |  | **High** risk of extubation failure | |  |  |  |  |  |  |  |  |  |  |  |  |  |  |  |
| Su/2012 | Multicenter (3 ICUs in China) | 406 | Patients received IMV for > 48 hrs and met the weaning criteria and passed a 2-hour SBT | | Pneumonia, postoperative respiratory failure, sepsis,COPD, heart failure, or other reasons | | NIV through a full facial mask using Bi-PAP device in a spontaneous mode to achieve an SpO_2_ ≥ 92%, | COT with a oxygen aerosol mask to maintain SpO_2_ ≥ 92% | 17.93± 0.64 | | | 18.40±0.68 | | Unavailable | | Unavailable | | within 72 hrs post-extubation | Reintubation and RF within 72 hrs after extubation, ICU mortality |
|  |  |  | The risk of extubation failure was **unclear** | |  |  |  |  |  |  |  |  |  |  |  |  |  |  |  |
| Vargas/2017 | Multicenter (six ICUs in French) | 143 | (1) Patients intubated for > 48 hrs and tolerated SBT for 2 hrs; and (2) patients with known or suspected  chronic respiratory disorders or those who tolerated SBT with hypercapnia (PaCO2 > 45 mmHg) | | Exacerbation of chronic respiratory disorder, pneumonia, cardiac failure, or other reasons | | NIV was delivered immediately after extubation using PSV+PEEP or Bi-PAP mode via a face mask to achieve a SpO_2_ > 90%. NIV was not used continuously but used for 1 h every 3 h, and a minimal duration of 6 h a day were needed. | COT immediately after extubation to maintain SpO_2_ ≥ 90% | 46(38–59) ^a^ | | | 45(35–62) ^a^ | | > 45 mHg | > 45 mHg | | | 48 hrs after extubation | Reintubation, RF within 48 hrs after extubation, ICU mortality, length of ICU stay |
|  |  |  | **High** risk of extubation failure | |  |  |  |  |  |  |  |  |  |  |  |  |  |  |  |
| Fernandez/2017 | Multicenter (4 ICUs in Spain) | 155 | Adult non-hypercapnic patients receiving IMV >12 hrs deemed ready for scheduled extubation after tolerance of SBT and were at high risk of extubation failure | | Unavailable | | HFOT after extubation was supplied by Optiflow device to achieve SpO_2_ of 92-95%. Flow was started at 40 L/min and was adjusted according to patients’ subjective tolerance. | COT after extubation was supplied either by nasal prongs or VM to achieve SpO_2_ of 92-95%. | 21 ± 8.8 | | | 21 ± 8.2 | Non-hypercapnic | | Non-hypercapnic | | | 24 hrs after extubation | Reintubation and RF within 72 hrs after extubation, ICU and inhospital mortality, length of ICU and inhospital stay |
|  |  |  | **High** risk of extubation failure | |  |  |  |  |  |  |  |  |  |  |  |  |  |  |  |
| Hernández/2016-L | Multicenter(7 ICUs in Spain) | 527 | Adult patients receiving IMV > 12 hrs deemed ready for scheduled extubation after tolerating SBT and met the criteria for low risk of reintubation | | Respiratory primary failure, nonrespiratory primary failure, surgery, and trauma | | HFOT was applied immediately after extubation by Optiflow device through nasal cannula to target SpO_2_ > 92%. Flow was initially set at 10 L/min titrated upward in 5 L/min steps. | COT was applied continuously through nasal cannula or nonrebreather facemask to maintain SpO_2_ > 92%. | 14 (9-16) | | 13 (9-17) | | 39 ±2.4 | | 38±2.9 | | | 24 hrs after extubation | Reintubation and RF within 72 hrs after extubation, ICU and inhospital mortality, length of ICU and inhospital stay |
|  |  |  | **Low** risk of extubation failure | |  |  |  |  |  |  |  |  |  |  |  |  |  |  |  |
| Maggiore/2014 | Multicenter (2 ICUs in Italy) | 105 | Patients mechanically ventilated for > 24 hrs and successfully passed SBT and had a PaO2/FiO2 ≤ 300 at  the end of the SBT | | Pneumonia, multiple trauma, atelectasis, shock, or other reasons | | Nasal HFOT was used immediately after extubation to obtain an SpO_2_ between 92% and 98%, the gas flow rate was 50 L/min | COT was delivered immediately after extubation by VM to obtain an SpO_2_ between 92% and 98% | 43 ± 14 ^a^ | | | 44 ± 16 ^a^ | | 34.7 ± 7.6 | | 36 ± 7.1 | | 48 hrs after extubation or up to ICU discharge | Reintubation and RF within 48 hrs after extubation, ICU mortality, length of ICU stay, and comfort score |
|  |  |  | The risk of extubation failure was **unclear** | |  |  |  |  |  |  |  |  |  |  |  |  |  |  |  |
| Song/2017 | Single-center (China) | 60 | Acute RF patients undergone IMV for > 48 hrs and met the weaning criteria and successfully passed the SBT and ready for extubation. | | Pneumonia, exacerbation of COPD, cardiogenic pulmonary edema, or other reasons | | HFOT were delivered immediately after extubation using PT101AZ device to obtain a SpO_2_ of 94–98%. The intial flow level was set at 60 L/min and was adjusted downward in 5 to 10 L/min. | COT was used, with air entrainment mask, to obtain a SpO_2_ of 94–98%. The FiO_2_ was set at 40%. | 12.87 ± 3.0 | | | 12.36 ±3.29 | | 41.5±6.7 | | 42.3±7.1 | | 24 hrs after extubation. | Reintubation within 24 hrs after extubation, and comfort score |
|  |  |  | **High** risk of extubation failure | |  |  |  |  |  |  |  |  |  |  |  |  |  |  |  |
| Hernández/2016-H | Multicenter(3 ICUs in Spain) | 604 | Adult patients receiving IMV > 12 hrs deemed ready for scheduled extubation after tolerating SBT and met the criteria for high risk of extubation failure | | Respiratory infection, exacerbated COPD, cardiologic disease, trauma, urgent surgery, or other reasons | | HFOT was applied immediately after extubation using Optiflow device through specific nasal  cannula to target SpO_2_ > 92%. Flow was initially set at 10 L/min and titrated upwards in 5 L/min steps. | NIV, with full face mask, was continuously delivered immediately after extubation using Bi-PAP ventilator to maintain SpO_2_ > 92%. | 16(13.8-22) | | | 16 (14-21) | | 41±2.2 | | 39 ±3.2 | | 24 hrs after extubation. | Reintubation and RF within 72 hrs after extubation, ICU and inhospital mortality, length of ICU and inhospital stay |
|  |  |  | **High** risk of extubation failure | |  |  |  |  |  |  |  |  |  |  |  |  |  |  |  |
| Jing/2019 | Single-center (China) | 42 | COPD patients intubated for exacerbation, with hypercapnia (PaCO2 >45 mmHg）at the time of extubation, and met the “pulmonary infection control window” criteria | | Exacerbation of COPD | | HFOT, with nasal cannulas, was applied immediately after extubation using Optiflow or AIRVO device at least 8 hrs/day to maintain SpO_2_ at 88–92%. | NIV with standard oral-nasal mask was used with VPAP III ST ventilator by using Bi-PAP mode at least 8 hrs/day to maintain SpO_2_ 88– 92%, | 11.8 ± 3.1 | | | 10.4 ± 2.5 | | 53.2±6.7 | | 53.7±8.6 | | 48 hrs after extubation | Reintubation and RF within 48 hrs after extubation, 28-day mortality, length of ICU stay, and comfort score |
|  |  |  | **High** risk of extubation failure | |  |  |  |  |  |  |  |  |  |  |  |  |  |  |  |
| Zhang/2018 | Single-center (China) | 45 | COPD patients treated with IMV and successfully passed a SBT and met the criteria of extubation | | Exacerbation of COPD | | HFOT, with nasal cannulas, was used after extubatoin with AIRVO device to maintain SpO_2_ at 88–92%. | NIV, with face mask, was used after extubatoin with Philips V60 ventilator by using Bi-PAP mode to maintain SpO_2_ at 88–92%. | 16.3 ± 1.4 | | | 16.7 ± 1.6 | | 42.9±7.7 | | 43.3±8.1 | | Study continued untill disappearance of signs of respiratory distress (and PaO2/FiO2 >300) last > 24 hrs | Reintubation in the ICU, 28-day mortality, length of ICU stay |
|  |  |  | **High** risk of extubation failure | |  |  |  |  |  |  |  |  |  |  |  |  |  |  |  |
| Thille/2019 | Multicenter (30 ICUs in France) | 641 | Adult patients intubated > 24 hrs in ICU and ready for extubation after a successful SBT, and were at high risk of extubation failure | | Acute RF, coma, shock, cardiac arrest, or other reasons | | HFOT plus NIV was used in this group. NIV was initiated immediately after extubation by using PSV +PEEP mode with a first session of > 4 hrs and minimal duration of > 12 hrs a day. Between NIV sessions, HFOT was delivered. The oxygenation target was to obtain SpO_2_ > 92%. | HFOT were continuously used after extubation > 48 hrsv with a flow of 50 L/min to obtain SpO_2_ > 92%. | 55 ± 20 ^a^ | | | 55 ± 17 ^a^ | | 40±9 | | 39±8 | | A minimum of 48 hrs following extubation. When there  were no signs of RF 48 hrs after extubation, treatment was stopped | Reintubation within 72 hrs after extubation, RF within 7 days after extubation, ICU and inhospital mortality, length of ICU stay |
|  |  |  | **High** risk of extubation failure | |  |  |  |  |  |  |  |  |  |  |  |  |  |  |  |

The data on APACHE II/SAPS II score and PaCO_2_ are presented as mean±SD or median (IQR), the unit of PaCO_2_ is mm Hg; ^a^ SAPS II score presented.

ICU intensive care unit; No. number; RF respiratory failure; IMV invasive mechanical ventilation; SBT spontaneous breathing trial; hrs hours; PaCO_2_ atrial partial pressure of carbon dioxide; FiO_2_ fraction of inspired oxygen; SpO_2_ oxygen saturation as measured by pulse oximetry; AECOPD acute exacerbation of chronic obstructive pulmony disease; ARDS acute respiratory distress syndrome; NIV noninvasive ventilation; HFOT high-flow oxygen therapy; COT conventional oxygen therapy; VM face mask; Bi-PAP bi-level postive airway pressure; PEEP postive end expiratory pressure; PSV pressure support ventilation; APACHE Acute Physiology and Chronic Health Evaluation ; SAPS simplified acute physiology score; EIG experimental intervention group; CIG control intervention group; SD standard deviation; IQR interquartile range

# Table S3. Detailed outcomes from the included studies

| First author /Publication year | Reintubation rate  (events/total) | | Post-extubation respiratory failure  (events/total) | | | Short-term mortality  (death/total) | | | Length of ICU stay (days)  [mean±SD/median(IQR)] | | Length of in-hospital stay(days)  [mean±SD/median(IQR)] | | | Comfort score  [mean±SD/median(IQR)] | |
| --- | --- | --- | --- | --- | --- | --- | --- | --- | --- | --- | --- | --- | --- | --- | --- |
|  | EIG | CIG | EIG | | CIG | EIG | | CIG | EIG | CIG | EIG | CIG | | EIG | CIG |
| Adiyeke/2016 | 3/25 | 5/25 | 3/25 | 19/25 | | 3/25 | 4/25 | | 5.2 ± 4.9 | 16.7 ± 7.7 | Unavailable | | Unavailable | Unavailable | Unavailable |
| Ferrer/2006 | 9/79 | 18/83 | 13/79 | 27/83 | | ICU mortality | | | 11 ± 8 | 13 ± 11 | 30 ± 23 | | 29 ± 18 | Unavailable | Unavailable |
|  |  |  |  |  |  | 2/79 | 12/83 | |  |  |  |  |  |  |  |
|  |  |  |  |  |  | In-hospital mortality | | |  |  |  |  |  |  |  |
|  |  |  |  |  |  | 13/79 | 19/83 | |  |  |  |  |  |  |  |
| Ferrer/2009 | 6/54 | 10/52 | 8/54 | 25/52 | | ICU mortality | | | 11 ± 13 | 10 ± 9 | 29 ± 27 | | 24 ± 17 | Unavailable | Unavailable |
|  |  |  |  |  |  | 3/54 | 4/52 | |  |  |  |  |  |  |  |
|  |  |  |  |  |  | In-hospital mortality | | |  |  |  |  |  |  |  |
|  |  |  |  |  |  | 6/54 | 11/52 | |  |  |  |  |  |  |  |
| Khilnani/2011 | 3/20 | 5/20 | Unavailable | Unavailable | | In-hospital mortality | | | 2.05 ± 2.18 | 1.55 ± 0.82 | 16.1 ± 6.29 | | 18.25 ± 7.91 | Unavailable | Unavailable |
|  |  |  |  |  |  | 0/20 | 1/20 | |  |  |  |  |  |  |  |
| Mohamed/2013 | 9/60 | 15/60 | Unavailable | Unavailable | | ICU mortality | | | 8.3 ± 3.1 | 11.6 ± 2.6 | Unavailable | | Unavailable | Unavailable | Unavailable |
|  |  |  |  |  |  | 4/60 | 10/60 | |  |  |  |  |  |  |  |
| Nava/2005 | 4/48 | 12/49 | Unavailable | Unavailable | | ICU mortality | | | 8.9 ± 5.7 | 11.6 ± 14.9 | 23.3 ± 16.4 | | 25.5 ± 21.4 | Unavailable | Unavailable |
|  |  |  |  |  |  | 3/48 | 9/49 | |  |  |  |  |  |  |  |
|  |  |  |  |  |  | In-hospital mortality | | |  |  |  |  |  |  |  |
|  |  |  |  |  |  | 6/48 | 9/49 | |  |  |  |  |  |  |  |
| Ornico/2013 | 1/20 | 7/18 | Unavailable | Unavailable | | In-hospital mortality | | | 16.8 ± 11.6 | 18.4 ± 12.2 | Unavailable | | Unavailable | Unavailable | Unavailable |
|  |  |  |  |  |  | 0/20 | 4/18 | |  |  |  |  |  |  |  |
| Su/2012 | 21/202 | 16/204 | 30/202 | 27/204 | | ICU mortality | | | Unavailable | Unavailable | Unavailable | | Unavailable | Unavailable | Unavailable |
|  |  |  |  |  |  | 3/202 | 2/204 | |  |  |  |  |  |  |  |
| Vargas/2017 | 6/71 | 13/72 | 6/71 | 20/72 | | ICU mortality | | | 4 (2-7) | 3 (2-6) | Unavailable | | Unavailable | Unavailable | Unavailable |
|  |  |  |  |  |  | 2/71 | 6/72 | |  |  |  |  |  |  |  |
| Fernandez/2017 | 9/78 | 12/77 | 16/78 | 21/77 | | ICU mortality | | | 12 (7-25) | 14 (9-17) | 27 (18-54) | | 27 (18-47) | Unavailable | Unavailable |
|  |  |  |  |  |  | 6/78 | 7/77 | |  |  |  |  |  |  |  |
|  |  |  |  |  |  | In-hospital mortality | | |  |  |  |  |  |  |  |
|  |  |  |  |  |  | 12/78 | 12/77 | |  |  |  |  |  |  |  |
| Hernández/2016-L | 13/264 | 32/263 | 22/264 | 38/263 | | ICU mortality | | | 6 (2-8) | 6 (2-9) | 11 (6-15) | | 12 (6-16) | Unavailable | Unavailable |
|  |  |  |  |  |  | 3/264 | 3/263 | |  |  |  |  |  |  |  |
|  |  |  |  |  |  | In-hospital mortality | | |  |  |  |  |  |  |  |
|  |  |  |  |  |  | 10/264 | 13/263 | |  |  |  |  |  |  |  |
| Maggiore/2014 | 2/53 | 11/52 | 4/53 | 18/52 | | ICU mortality | | | 11.7 ± 10.2 | 10.4 ± 8.5 | Unavailable | | Unavailable | 2.7 ± 2.4 | 4.5 ± 3.3 |
|  |  |  |  |  |  | 6/53 | 5/52 | |  |  |  |  |  |  |  |
| Song/2017 | 1/30 | 3/30 | Unavailable | Unavailable | | Unavailable | Unavailable | | Unavailable | Unavailable | Unavailable | | Unavailable | 3 (2-3.5) | 5 (4.7-6) |
| Hernández/2016-H | 66/290 | 60/314 | 78/290 | 125/314 | | ICU mortality | | | 3 (2-7) | 4 (2-9) | 23 (14-46) | | 26 (16-37) | Unavailable | Unavailable |
|  |  |  |  |  |  | 19/290 | 18/314 | |  |  |  |  |  |  |  |
|  |  |  |  |  |  | In-hospital mortality | | |  |  |  |  |  |  |  |
|  |  |  |  |  |  | 59/290 | 56/314 | |  |  |  |  |  |  |  |
| Jing/2019 | 2/22 | 1/20 | 3/22 | 1/20 | | 28-days mortaity | | | 8.5 ± 3.5 | 9.4 ± 4.8 | Unavailable | | Unavailable | 3.6 ± 1.9 | 5.2 ± 2.3 |
|  |  |  |  |  |  | 5/22 | 5/20 | |  |  |  |  |  |  |  |
| Zhang/2018 | 2/21 | 1/24 | Unavailable | Unavailable | | 28-days mortaity | | | 13.7 ± 0.8 | 15.2 ± 0.5 | Unavailable | | Unavailable | Unavailable | Unavailable |
|  |  |  |  |  |  | 1/21 | 1/24 | |  |  |  |  |  |  |  |
| Thille/2019 | 30/339 | 47/302 | 70/339 | 88/302 | | ICU mortality | | | 12 (7-19) | 11 (7-19) | 25 (15-42) | | 23 (15-39) | Unavailable | Unavailable |
|  |  |  |  |  |  | 21/339 | 26/302 | |  |  |  |  |  |  |  |
|  |  |  |  |  |  | In-hospital mortality | | |  |  |  |  |  |  |  |
|  |  |  |  |  |  | 54/339 | 46/302 | |  |  |  |  |  |  |  |

EIG intervention group; CIG control intervention group; ICU intensive care medicine; SD standard deviation; IQR interquartile range

# Table S4. Sensitivity analyses for the primary outcomes

| **Comparisons** | **After excluding trials with low or unclear risk of extubation failure** | | | | | | | | **After excluding trials with PaCO_2_ > 45 mmHg at the end of SBT** | | | | |
| --- | --- | --- | --- | --- | --- | --- | --- | --- | --- | --- | --- | --- | --- |
|  | No. of RCTs | | | Direct estimate | | Indirect estimate | NMA estimate | No. of RCTs | | | Direct estimate | Indirect estimate | NMA estimate |
| Re-intubation rate (RR with 95%CI); Test for inconsistency in entire network: *P* = 0.823 | | | | | | | | | Test for inconsistency in entire network: *P* = 0.517 | | | | |
| NIV vs. COT | | 8 | 0.50 (0.35, 0.71) | | 0.56 (0.25, 1.26) | | 0.51 (0.37, 0.70) | | 4 | 0.47 (0.30, 0.75) | | 0.37 (0.03, 0.65) | 0.43 (0.30, 0.61) |
| HFOT vs. COT | | 2 | 0.67 (0.32, 1.44) | | 0.61 (0.38, 0.97) | | 0.63 (0.42, 0.93) | | 4 | 0.45 (0.29, 0.71) | | 0.57 (0.33, 0.99) | 0.50 (0.35, 0.70) |
| NIV vs. HFOT | | 3 | 0.82 (0.61, 1.12) | | 0.74 (0.32, 1.71) | | 0.81 (0.61, 1.08) | | 2 | 0.83 (0.61, 1.13) | | 1.05 (0.55, 2.01) | 0.87 (0.66, 1.15) |
| HFOT+NIV vs. HFOT | | 1 | 0.57 (0.37, 0.87) | | NE | | 0.57 (0.37, 0.87) | | 1 | 0.57 (0.37, 0.87) | | NE | 0.57 (0.37, 0.87) |
| Short-term mortality (RR with 95%CI); Test for inconsistency in entire network: *P* = 0.342 | | | | | | | | | Test for inconsistency in entire network: *P* = 0.437 | | | | |
| NIV vs. COT | | 8 | 0.57 (0.39, 0.84) | | 0.88 (0.40, 1.96) | | 0.62 (0.44, 0.88) | | 4 | 0.60 (0.38, 0.97) | | 0.82 (0.45, 1.47) | 0.68 (0.47, 0.98) |
| HFOT vs. COT | | 1 | 0.99 (0.47, 2.06) | | 0.64 (0.39, 1.05) | | 0.73 (0.49, 1.11) | | 3 | 0.93 (0.57, 1.52) | | 0.69 (0.39, 1.22) | 0.82 (0.57, 1.19) |
| NIV vs. HFOT | | 3 | 0.89 (0.65, 1.22) | | 0.58 (0.25, 1.33) | | 0.85 (0.63, 1.13) | | 2 | 0.88 (0.63, 1.22) | | 0.65 (0.33, 1.28) | 0.83 (0.62, 1.11) |
| HFOT+NIV vs. HFOT | | 1 | 1.05 (0.73, 1.50) | | NE | | 1.05 (0.73, 1.50) | | 1 | 1.05 (0.73, 1.50) | | NE | 1.05 (0.73, 1.50) |

RCTs randomized controlled trials; NMA network meta-analysis; SBT spontaneous breathing trial; NIV noninvasive ventilation; HFOT high-flow oxygen therapy; COT conventional oxygen therapy; RR risk ratio; CI confidence interval; NE not estimable.

# Table S5. Sensitivity analyses for the SCURA statistics

| **Outcomes** | **After excluding trials with low or unclear risk of extubation failure** | | | | **After excluding trials with PaCO_2_ > 45 mmHg at the end of SBT** | | | | |
| --- | --- | --- | --- | --- | --- | --- | --- | --- | --- |
|  | **COT** | **NIV** | **HFOT** | **HFOT+NIV** |  | **COT** | **NIV** | **HFOT** | **HFOT+NIV** |
| Re-intubation rate | 0.4% | 67.1% | 35.7% | 96.8% | 0.0% | | 63.3% | 38.8% | 97.9% |
| Short-term mortality | 8.7% | 89.4% | 55.9% | 46.0% | 15.3% | | 90.4% | 52.4% | 41.9% |

SBT spontaneous breathing trial; NIV noninvasive ventilation; HFOT high-flow oxygen therapy; COT conventional oxygen therapy; PaCO_2_ atrial partial pressure of carbon dioxide

#
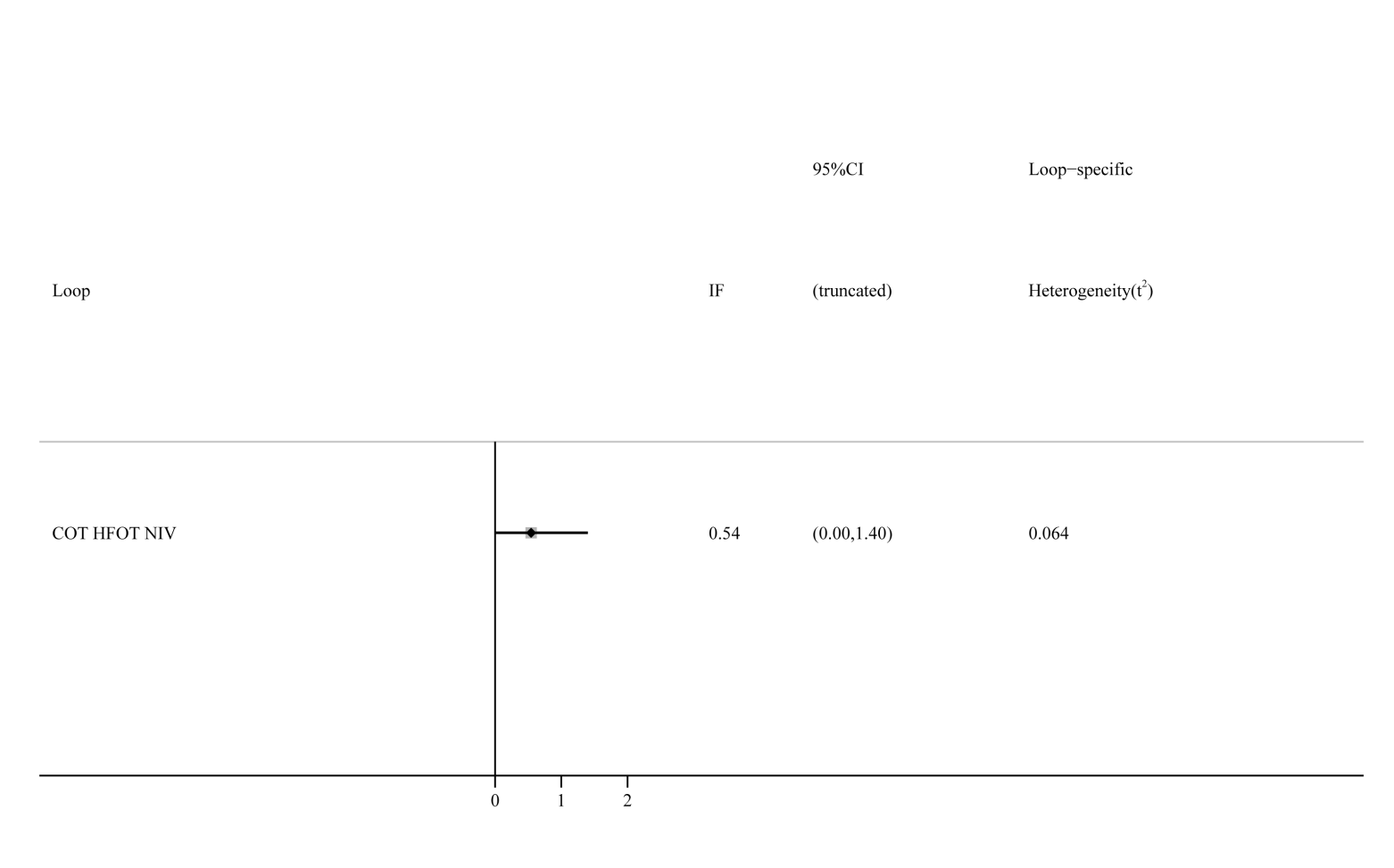
Figure S1. Inconsistency analysis for the re-intubation rate

NIV noninvasive ventilation; HFOT high-flow oxygen therapy; COT conventional oxygen therapy; CI confidence interval; IF inconsistency factor

#
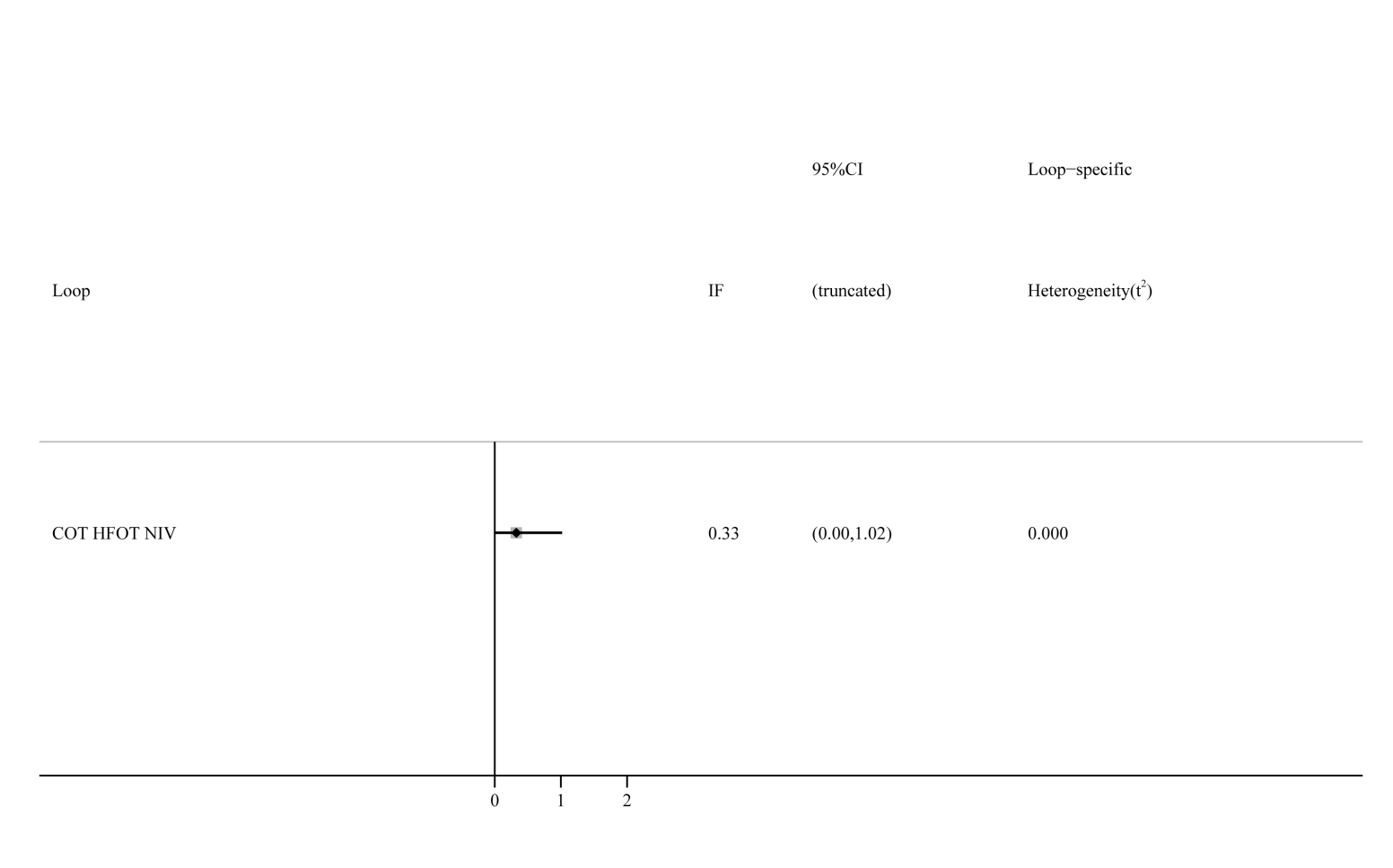
Figure S2. Inconsistency analysis for the short-term mortality

NIV noninvasive ventilation; HFOT high-flow oxygen therapy; COT conventional oxygen therapy; CI confidence interval; IF inconsistency factor

# Figure S3. Network geometry for the re-intubation rate


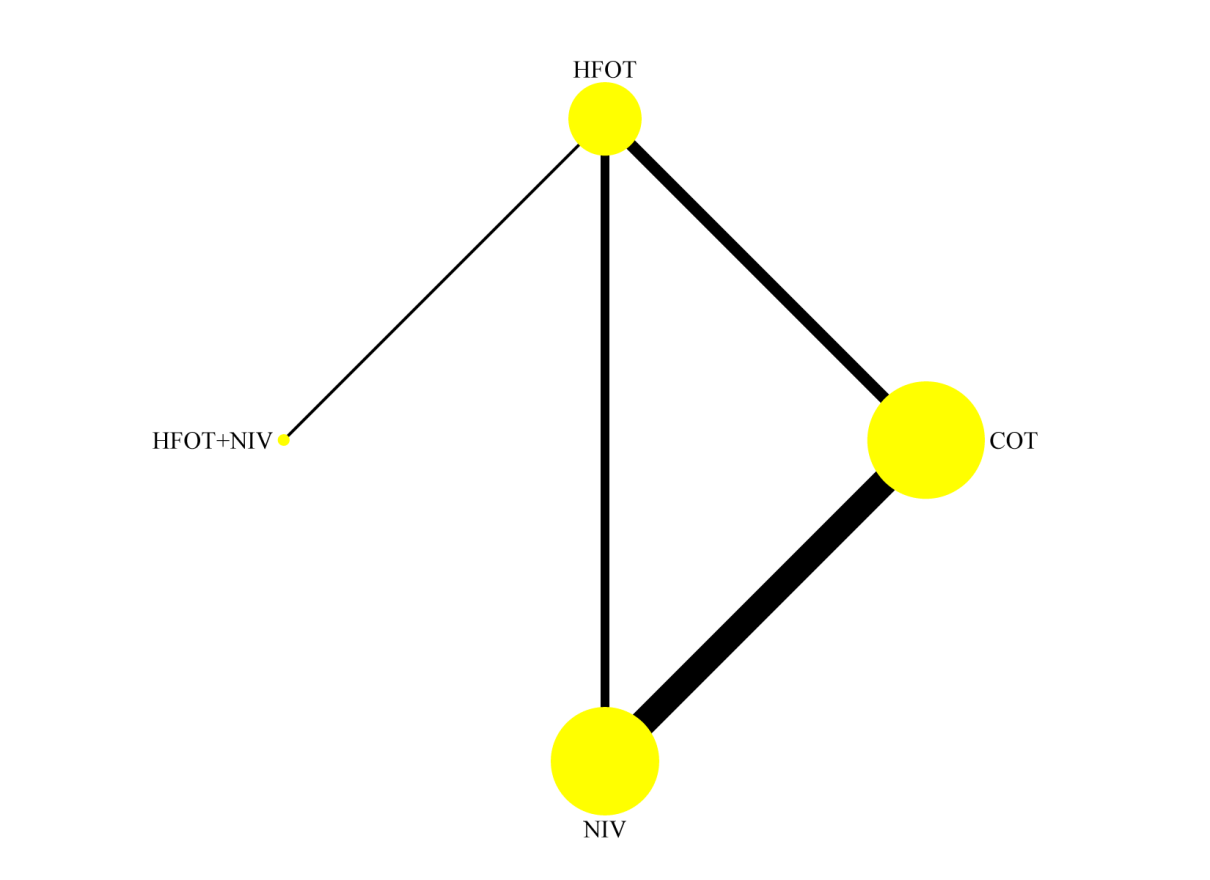


The size of the node was proportional to the number of trials that included in each method, and the thickness of the lines was proportional to the number of direct comparisons.

NIV noninvasive ventilation; HFOT high-flow oxygen therapy; COT conventional oxygen therapy

#
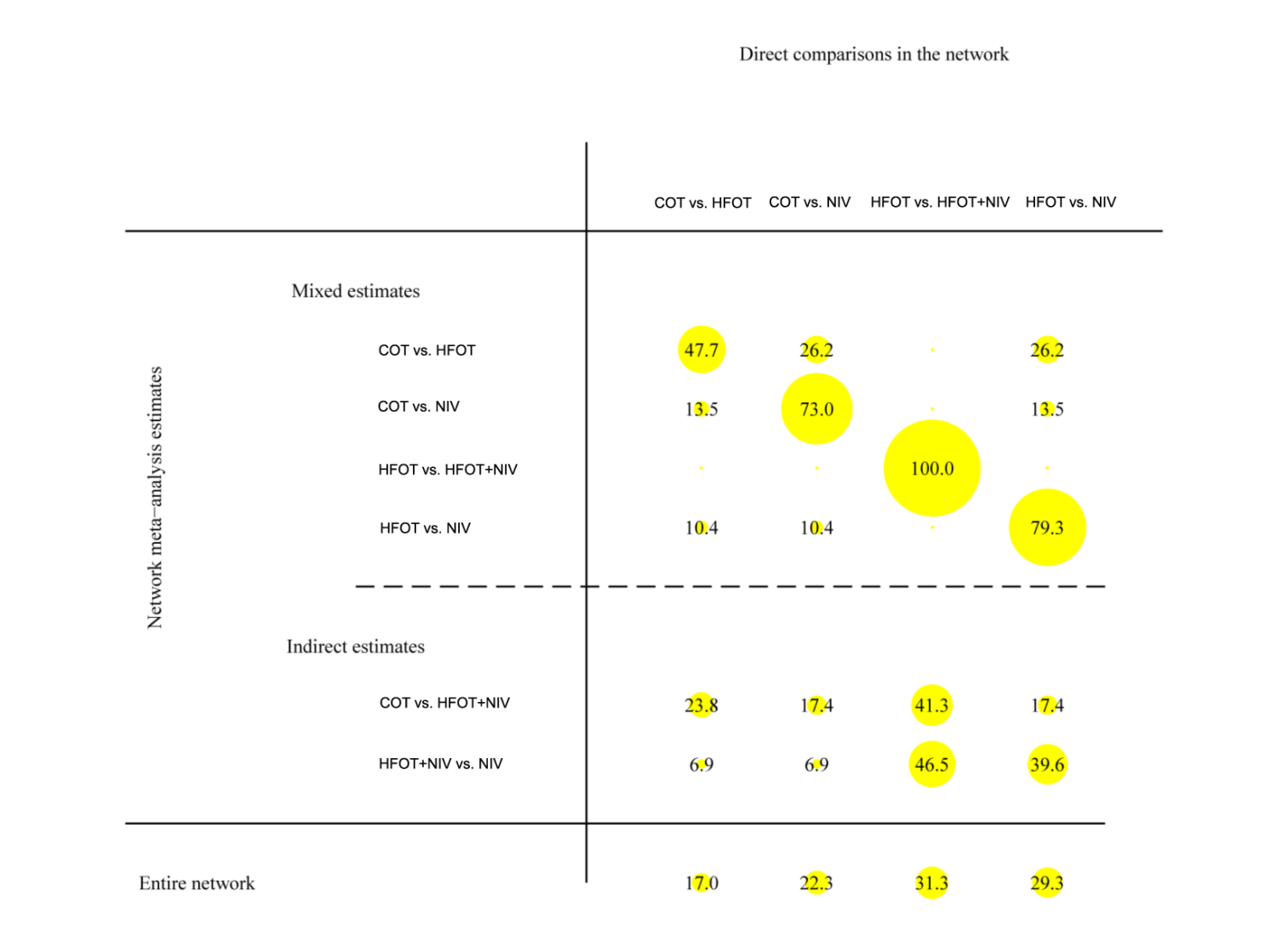
Figure S4. Weight contribution matrix for the re-intubation rate

NIV noninvasive ventilation; HFOT high-flow oxygen therapy; COT conventional oxygen therapy

# Figure S5. Network geometry for the short-term mortality


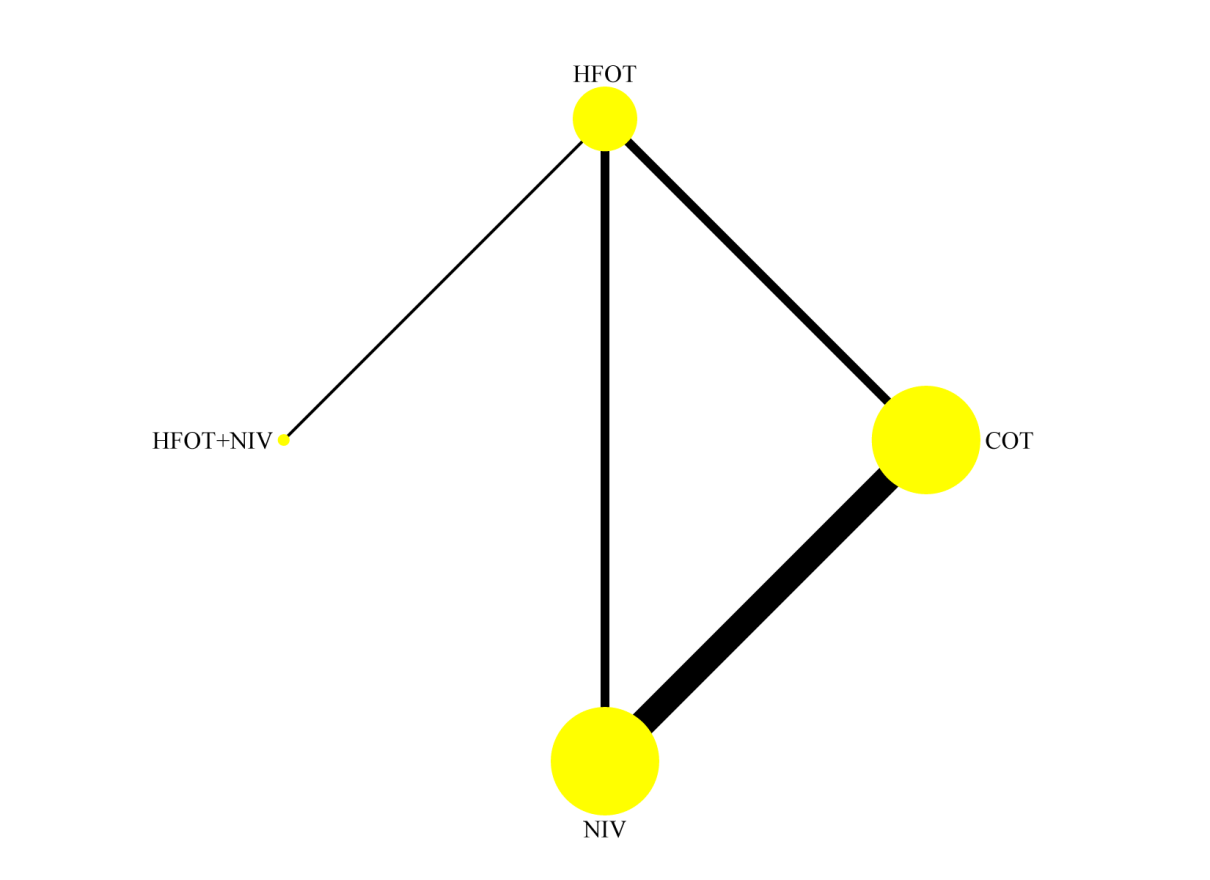


The size of the node was proportional to the number of trials that included in each method, and the thickness of the lines was proportional to the number of direct comparisons.

NIV noninvasive ventilation; HFOT high-flow oxygen therapy; COT conventional oxygen therapy

#
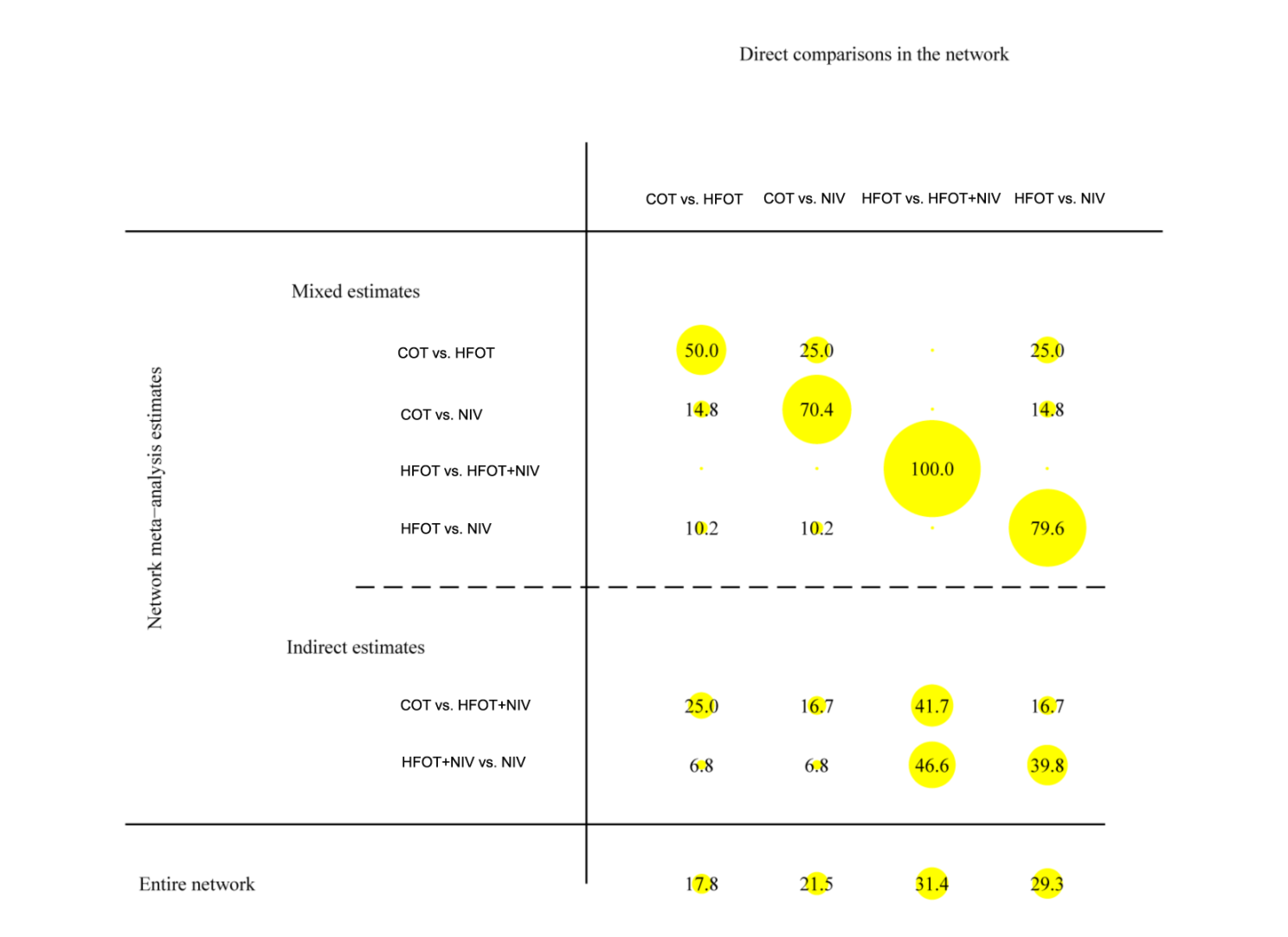
Figure S6. Weight contribution matrix for the short-term mortality

NIV noninvasive ventilation; HFOT high-flow oxygen therapy; COT conventional oxygen therapy

#
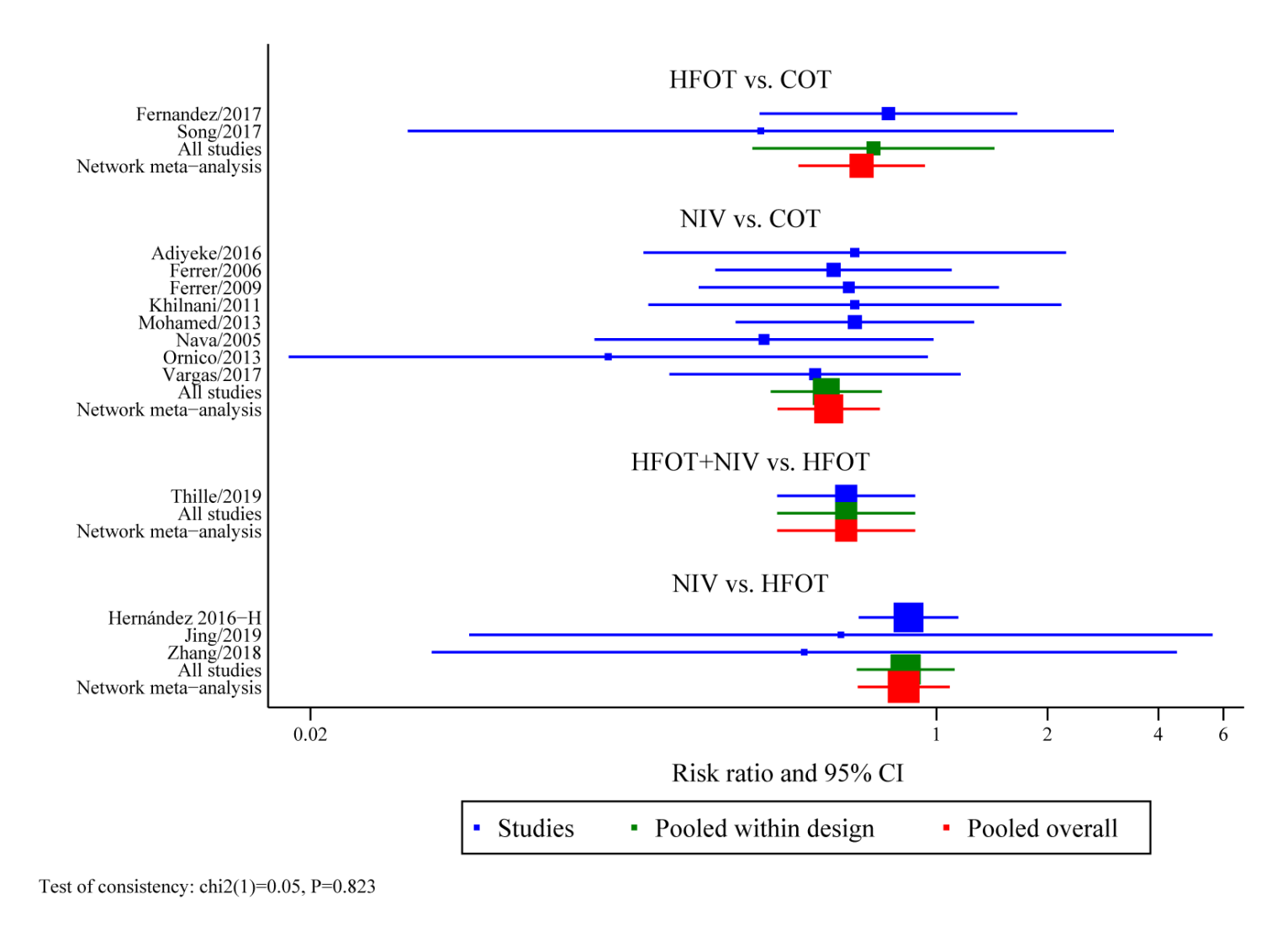
Figure S7. Forest plot of sensitivity analysis exclusively including trials with high risk of extubation failure for the re-intubation rate

NIV noninvasive ventilation; HFOT high-flow oxygen therapy; COT conventional oxygen therapy; CI confidence interval

#
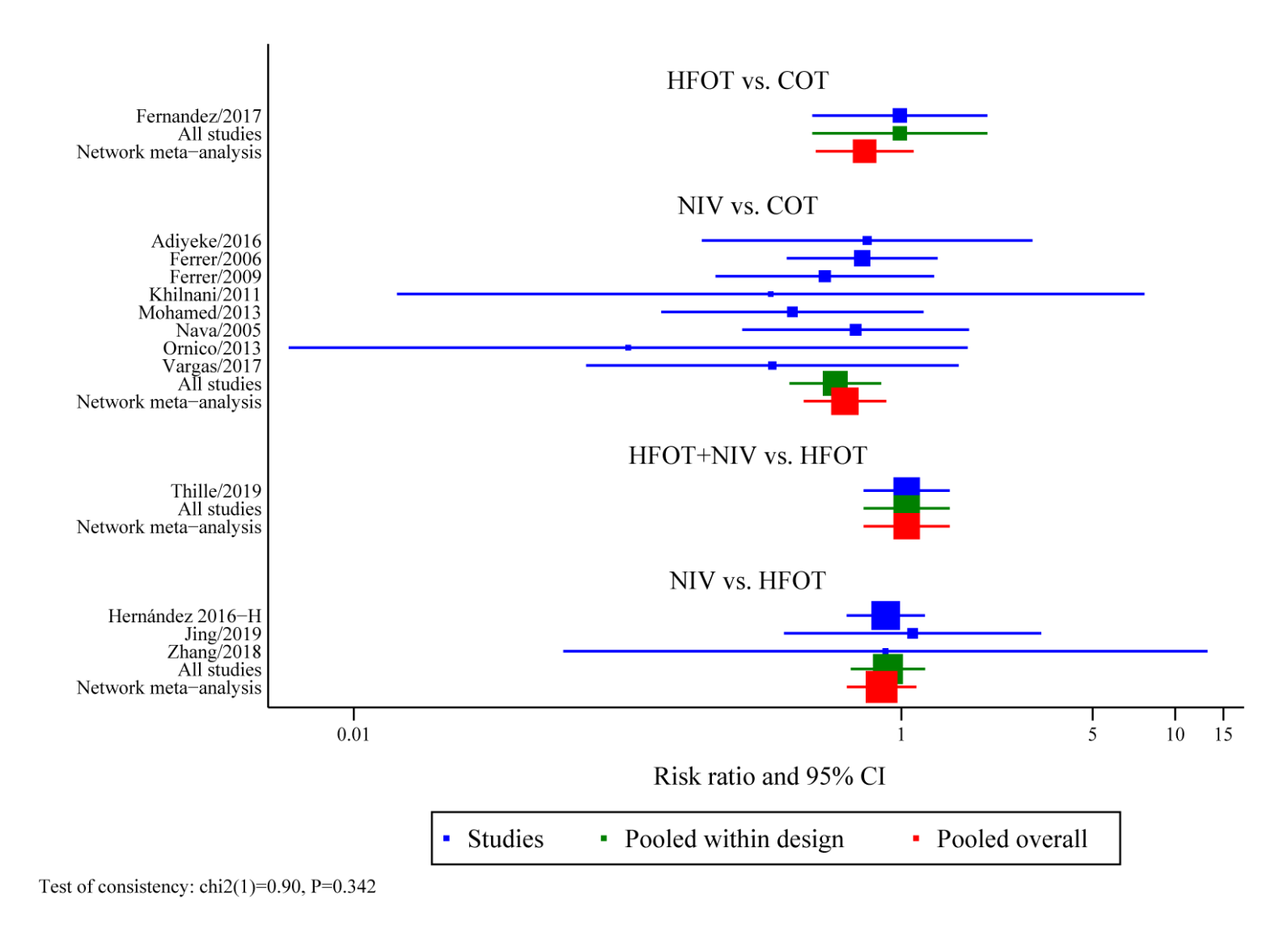
Figure S8. Forest plot of sensitivity analysis exclusively including trials with high risk of extubation failure for the short-term mortality

NIV noninvasive ventilation; HFOT high-flow oxygen therapy; COT conventional oxygen therapy; CI confidence interval

#
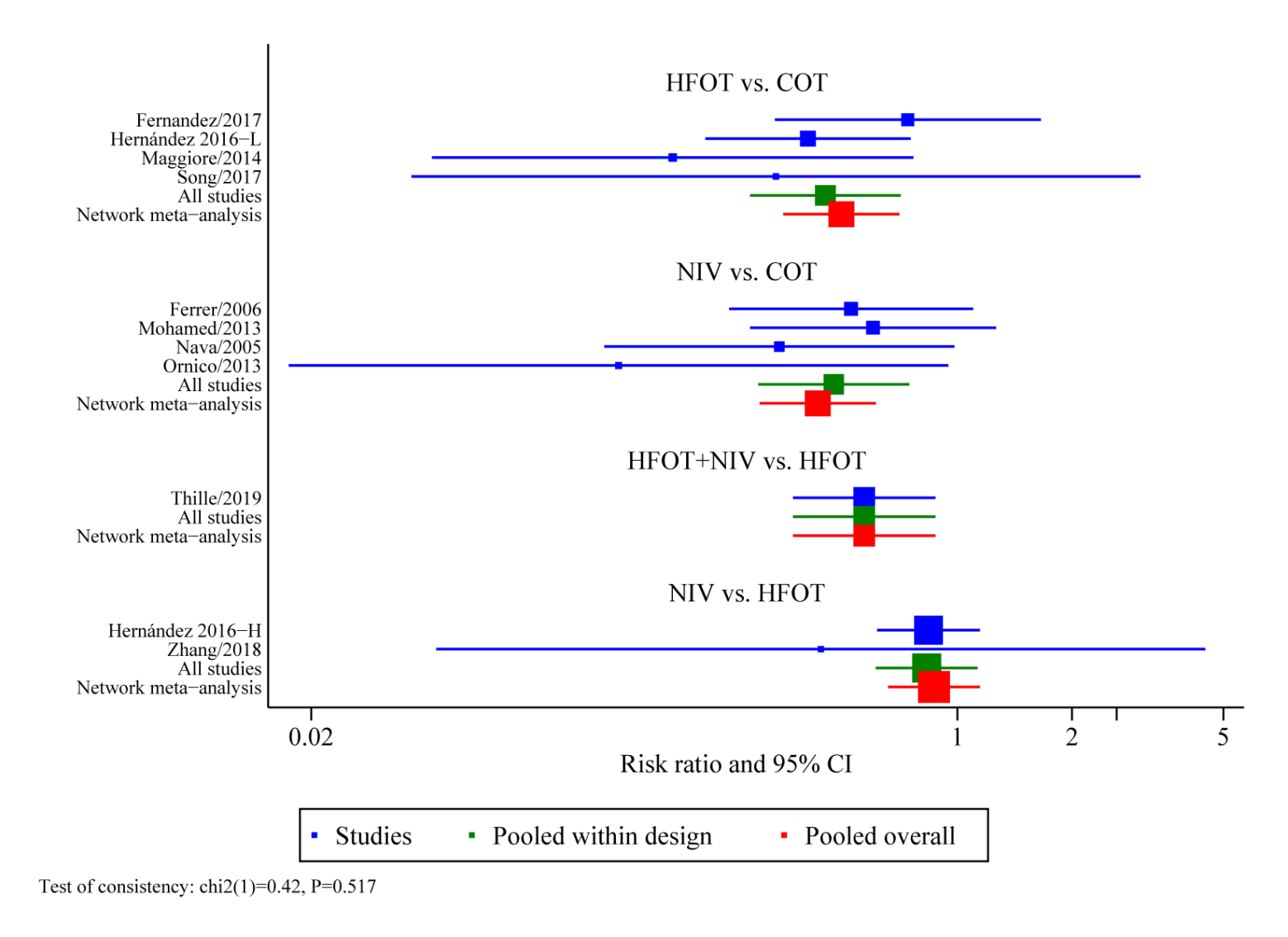
Figure S9. Forest plot of sensitivity analysis exclusively including trials with PaCO_2_ < 45 mmHg at the end of SBT for the re-intubation rate

NIV noninvasive ventilation; HFOT high-flow oxygen therapy; COT conventional oxygen therapy; CI confidence interval; PaCO_2_ atrial partial pressure of carbon dioxide; SBT spontaneous breathing trial

#
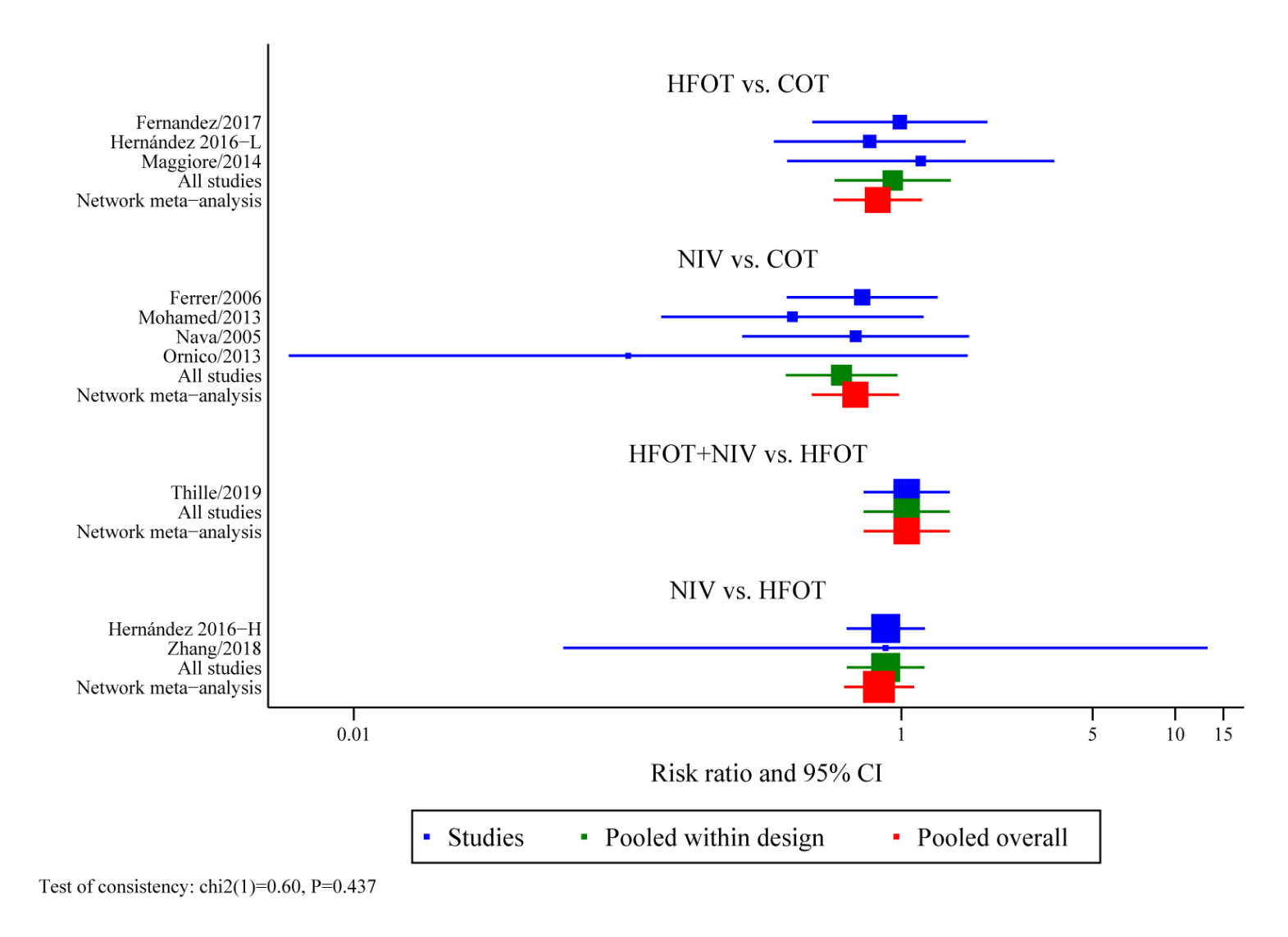
Figure S10. Forest plot of sensitivity analysis exclusively including trials with PaCO_2_ < 45 mmHg at the end of SBT for the short-term mortality

NIV noninvasive ventilation; HFOT high-flow oxygen therapy; COT conventional oxygen therapy; CI confidence interval; PaCO_2_ atrial partial pressure of carbon dioxide; SBT spontaneous breathing trial

#
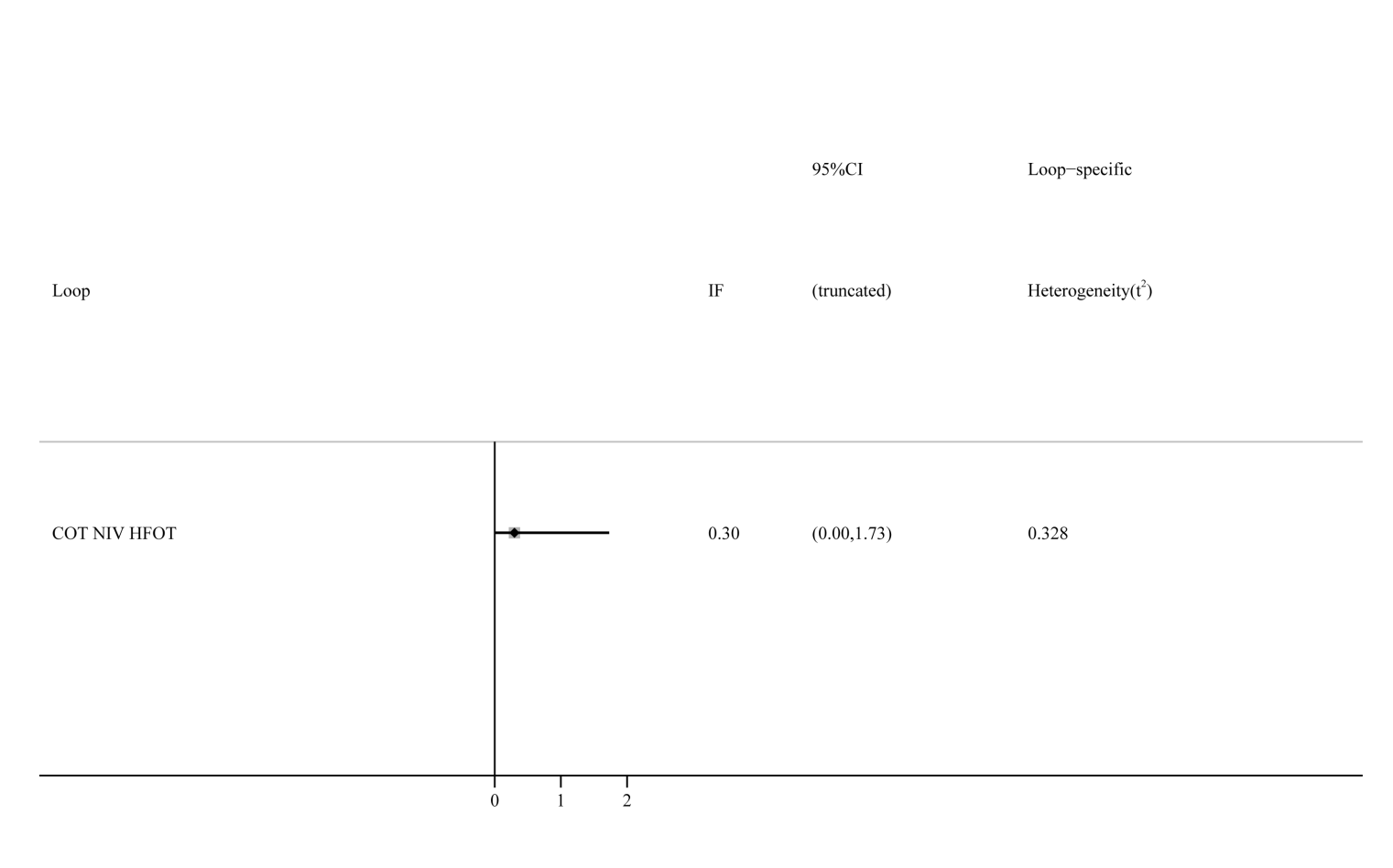
Figure S11. Inconsistency analysis for the post-extubation respiratory failure

NIV noninvasive ventilation; HFOT high-flow oxygen therapy; COT conventional oxygen therapy; CI confidence interval; IF inconsistency factor

#
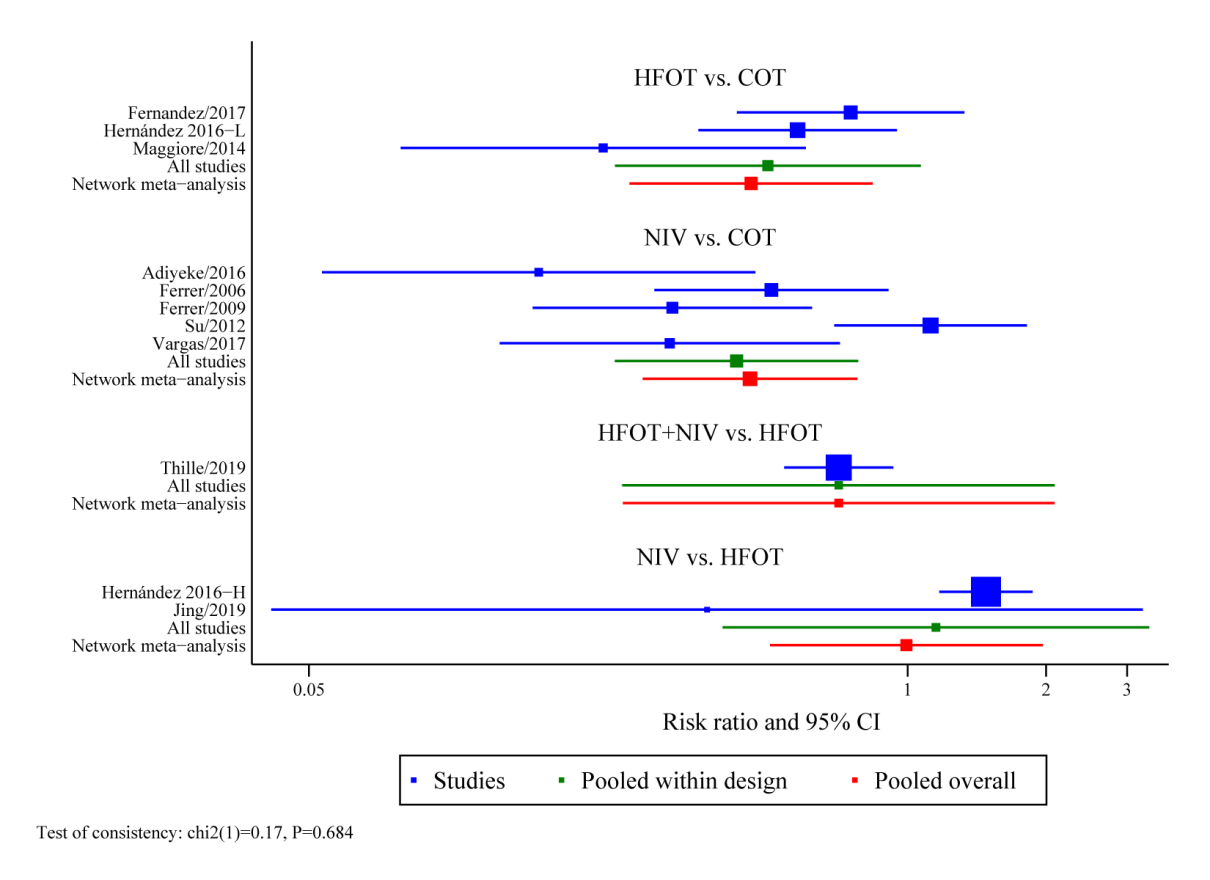
Figure S12. Forest plot of network meta-analysis for the post-extubation respiratory failure

NIV noninvasive ventilation; HFOT high-flow oxygen therapy; COT conventional oxygen therapy; CI confidence interval

# Figure S13. Inconsistency analysis for the length of ICU stay

ICU intensive care unit; NIV noninvasive ventilation; HFOT high-flow oxygen therapy; COT conventional oxygen therapy; CI confidence interval; IF inconsistency factor


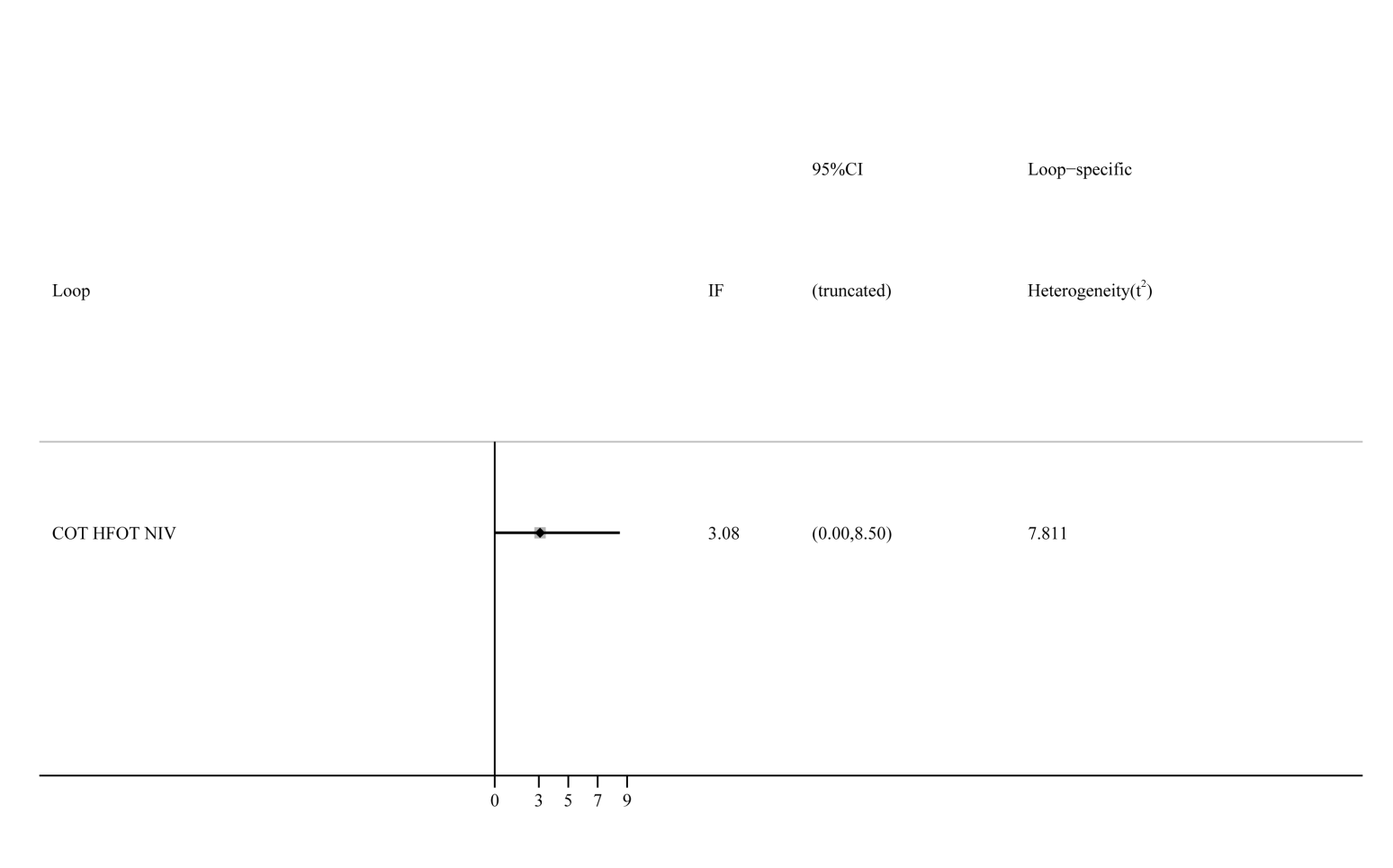


#
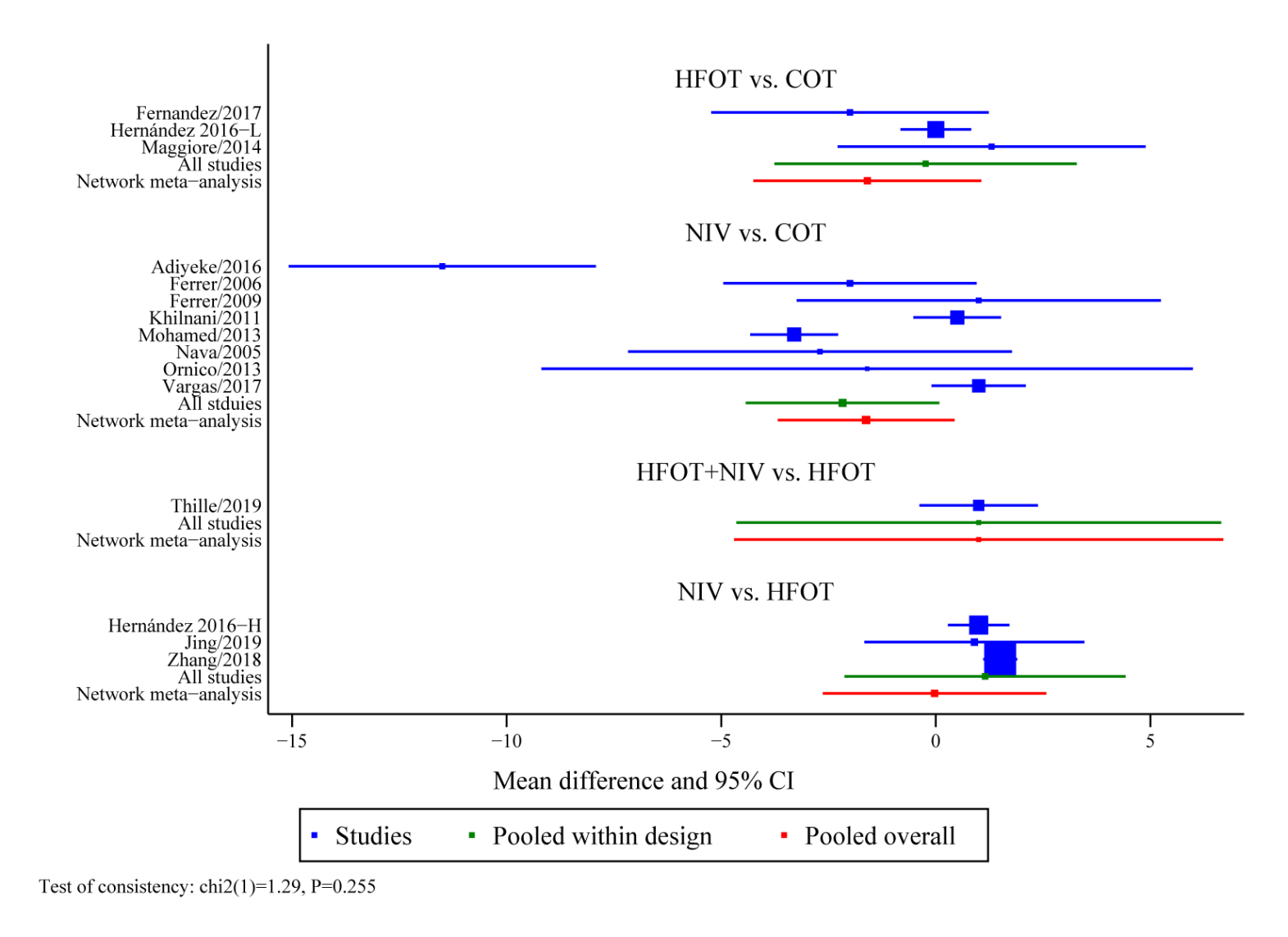
Figure S14. Forest plot of network meta-analysis for the length of ICU stay

ICU intensive care unit; NIV noninvasive ventilation; HFOT high-flow oxygen therapy; COT conventional oxygen therapy; CI confidence interval

#
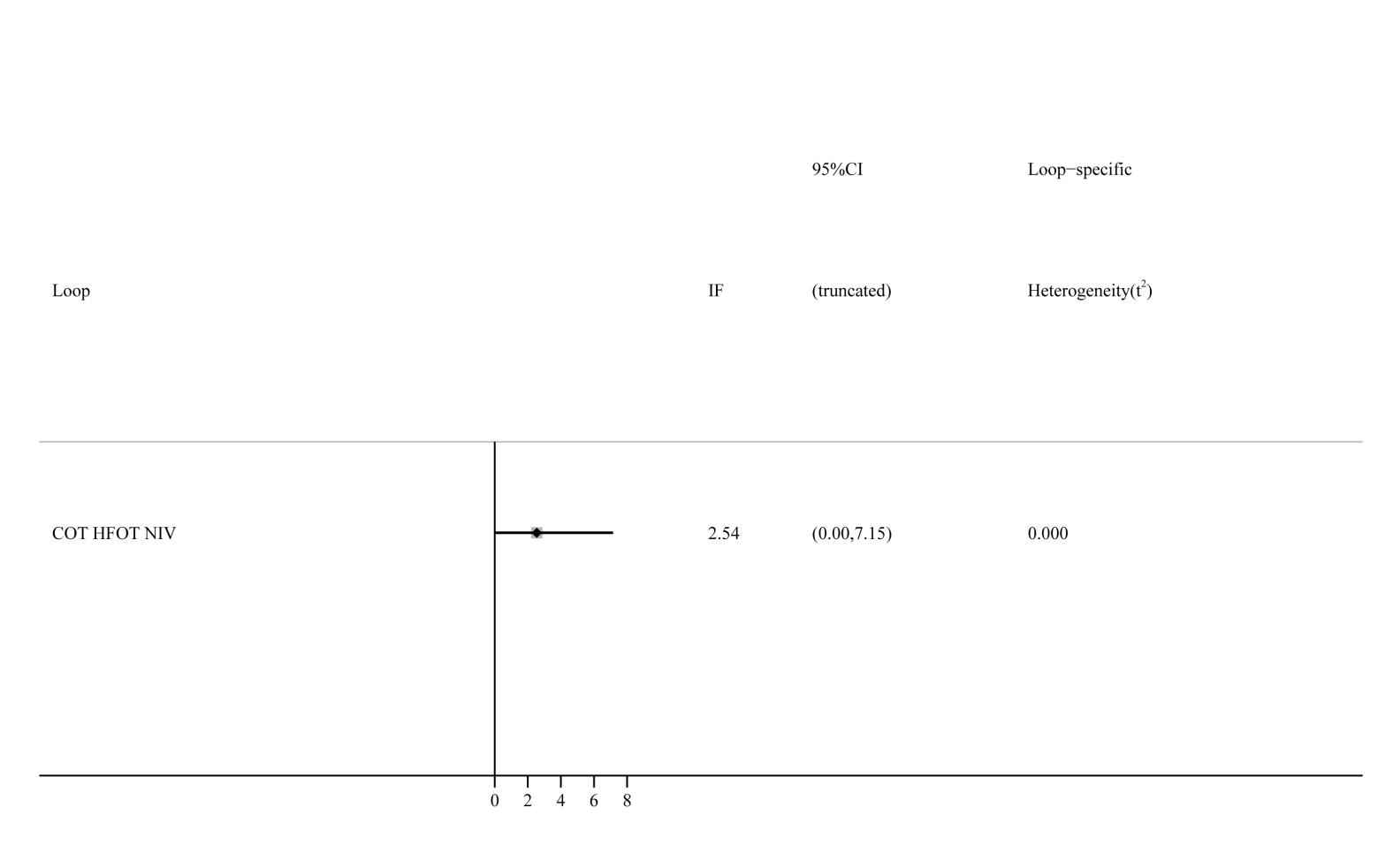
Figure S15. Inconsistency analysis for the length of in-hospital stay

NIV noninvasive ventilation; HFOT high-flow oxygen therapy; COT conventional oxygen therapy; CI confidence interval; IF inconsistency factor

# Figure S16. Forest plot of network meta-analysis for the length of in-hospital stay

NIV noninvasive ventilation; HFOT high-flow oxygen therapy; COT conventional oxygen therapy; CI confidence interval


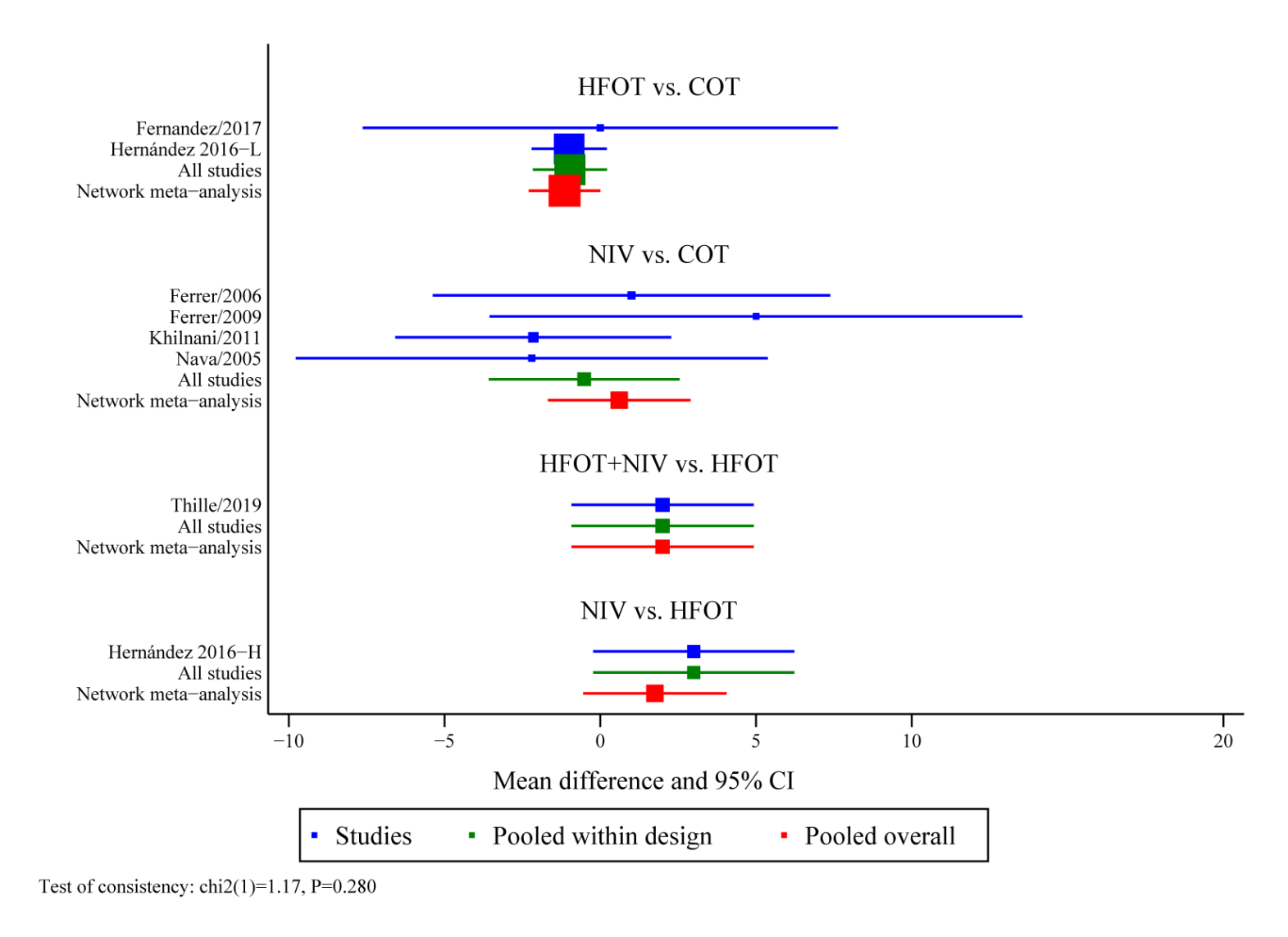


# Figure S17. Network geometry for the post-extubation respiratory failure


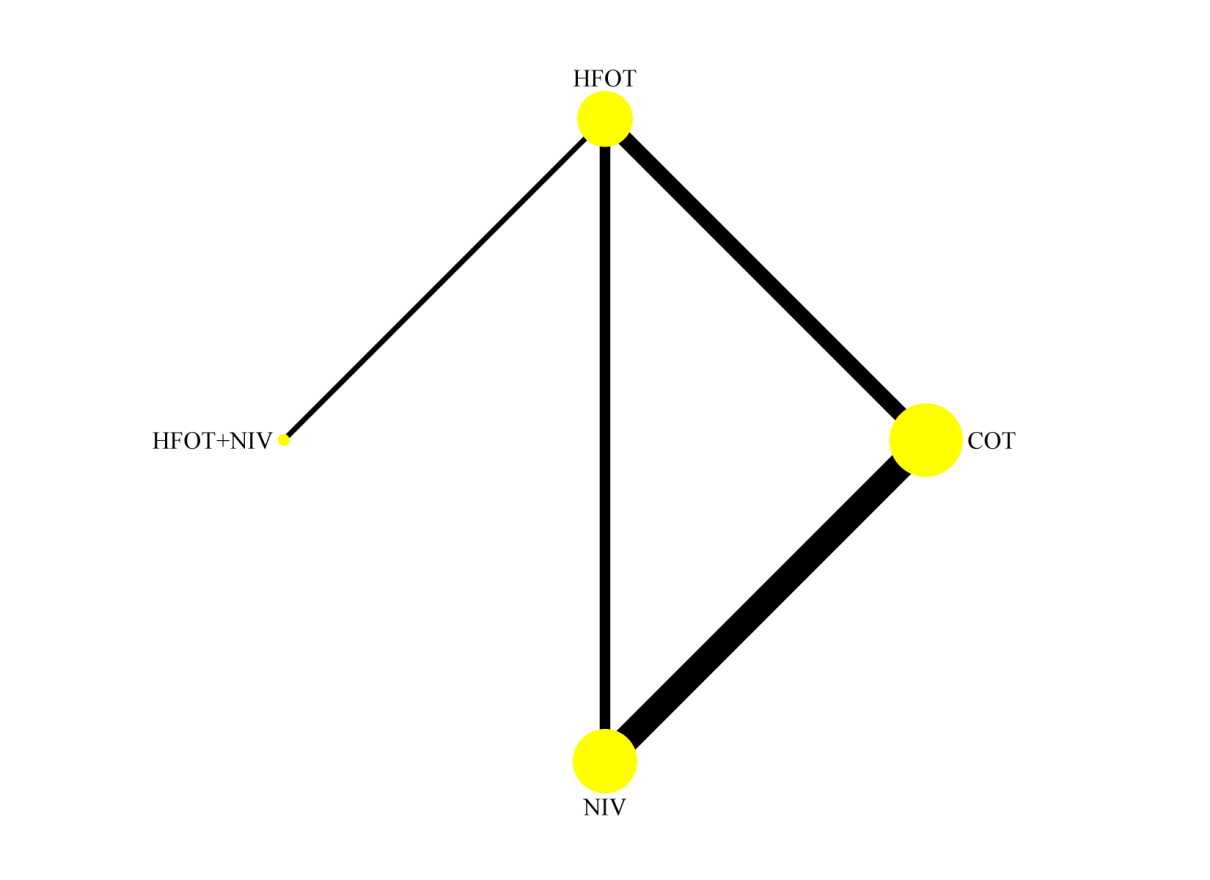


The size of the node was proportional to the number of trials that included in each method, and the thickness of the lines was proportional to the number of direct comparisons.

NIV noninvasive ventilation; HFOT high-flow oxygen therapy; COT conventional oxygen therapy

#
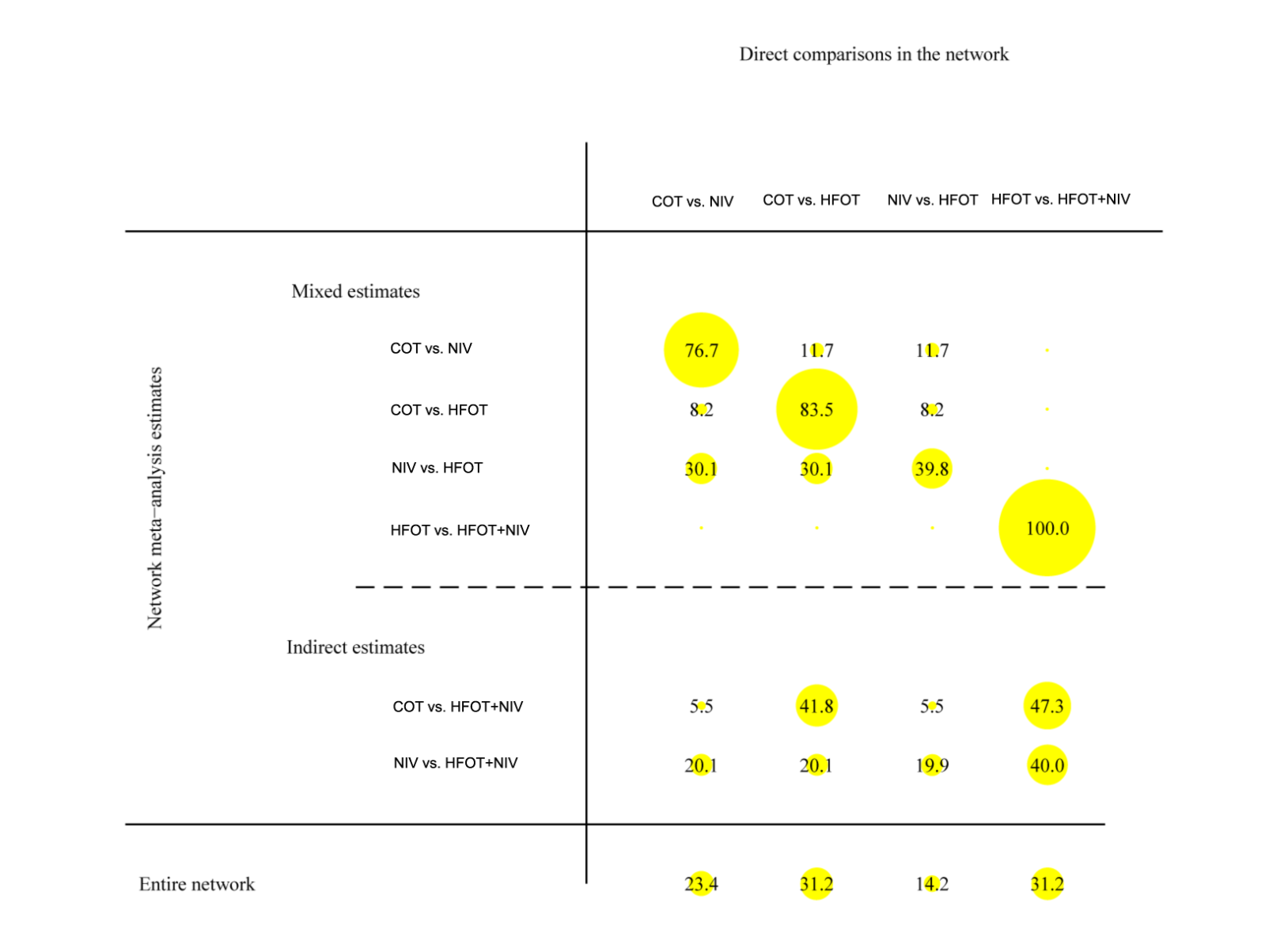
Figure S18. Weight contribution matrix for the post-extubation respiratory failure

NIV noninvasive ventilation; HFOT high-flow oxygen therapy; COT conventional oxygen therapy

# Figure S19. Network geometry for the length of ICU stay


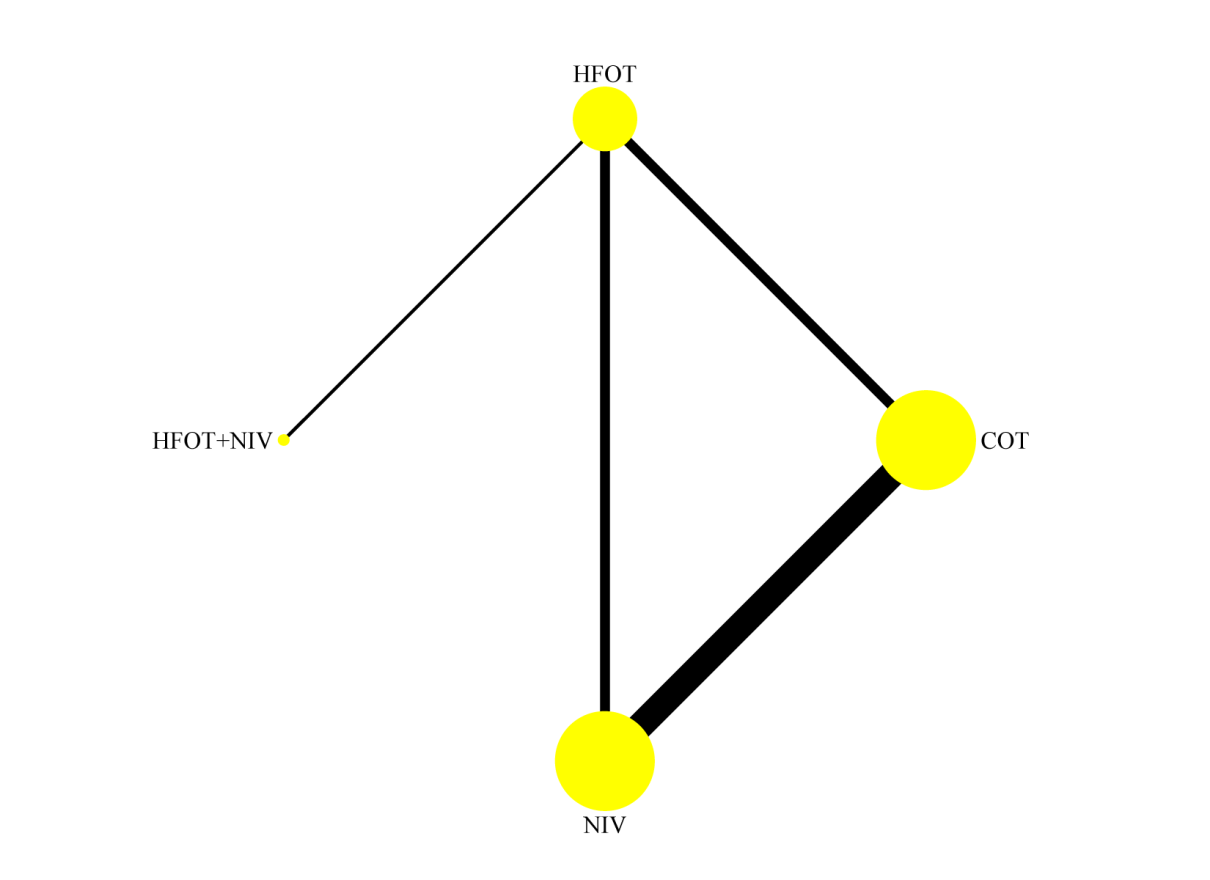


The size of the node was proportional to the number of trials that included in each method, and the thickness of the lines was proportional to the number of direct comparisons.

ICU intensive care unit; NIV noninvasive ventilation; HFOT high-flow oxygen therapy; COT conventional oxygen therapy

#
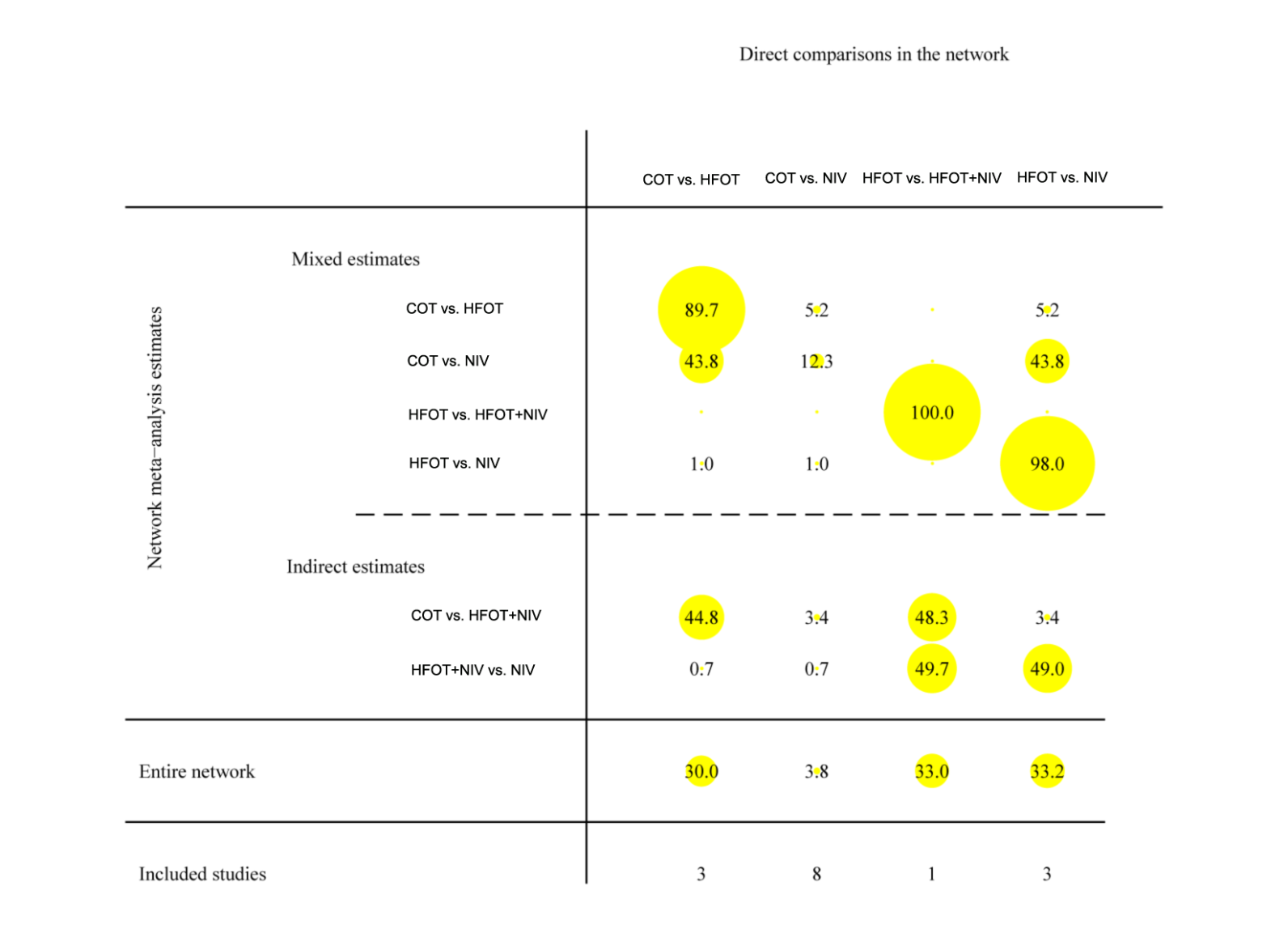
Figure S20. Weight contribution matrix for the length of ICU stay

ICU intensive care unit; NIV noninvasive ventilation; HFOT high-flow oxygen therapy; COT conventional oxygen therapy

# Figure S21. Network geometry for the length of in-hospital stay


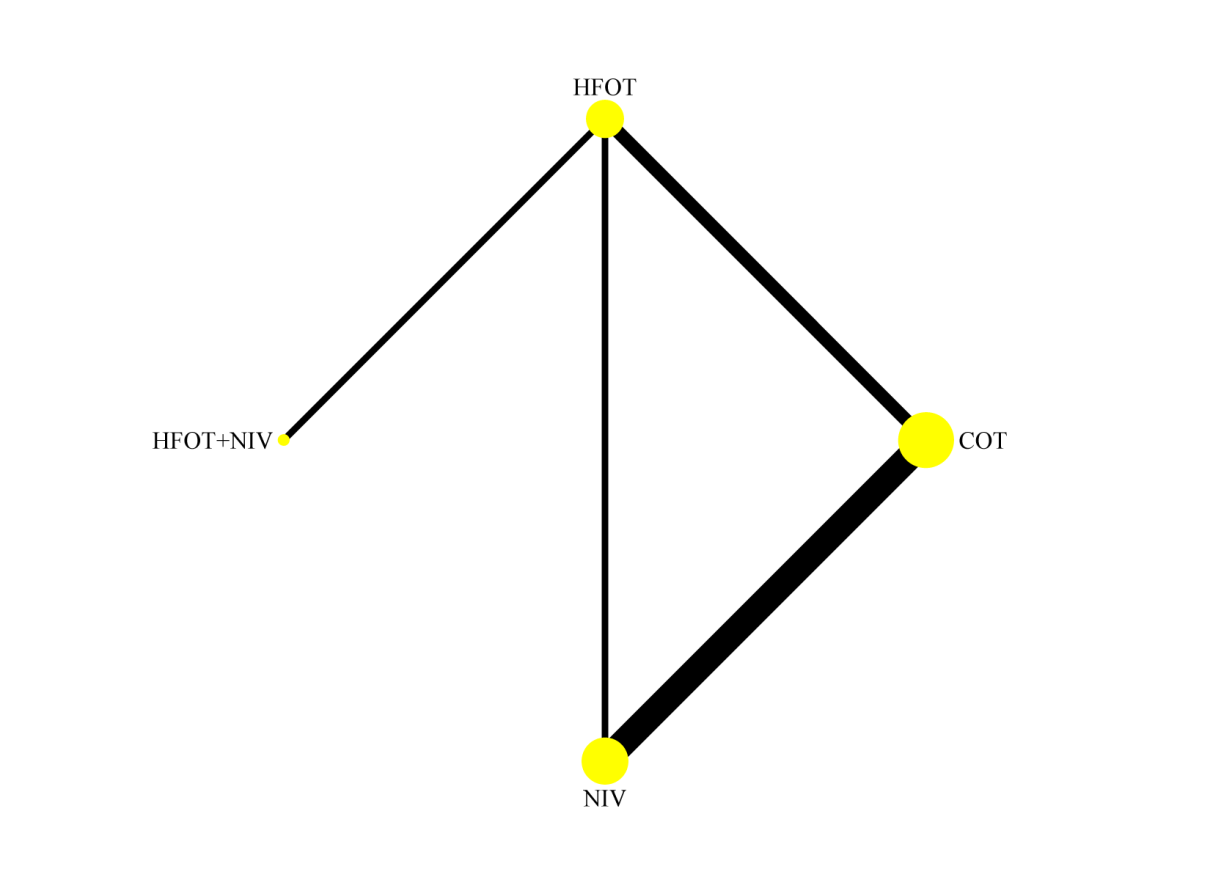


The size of the node was proportional to the number of trials that included in each method, and the thickness of the lines was proportional to the number of direct comparisons.

NIV noninvasive ventilation; HFOT high-flow oxygen therapy; COT conventional oxygen therapy

# Figure S22. Weight contribution matrix for the length of in-hospital stay

NIV noninvasive ventilation; HFOT high-flow oxygen therapy; COT conventional oxygen therapy
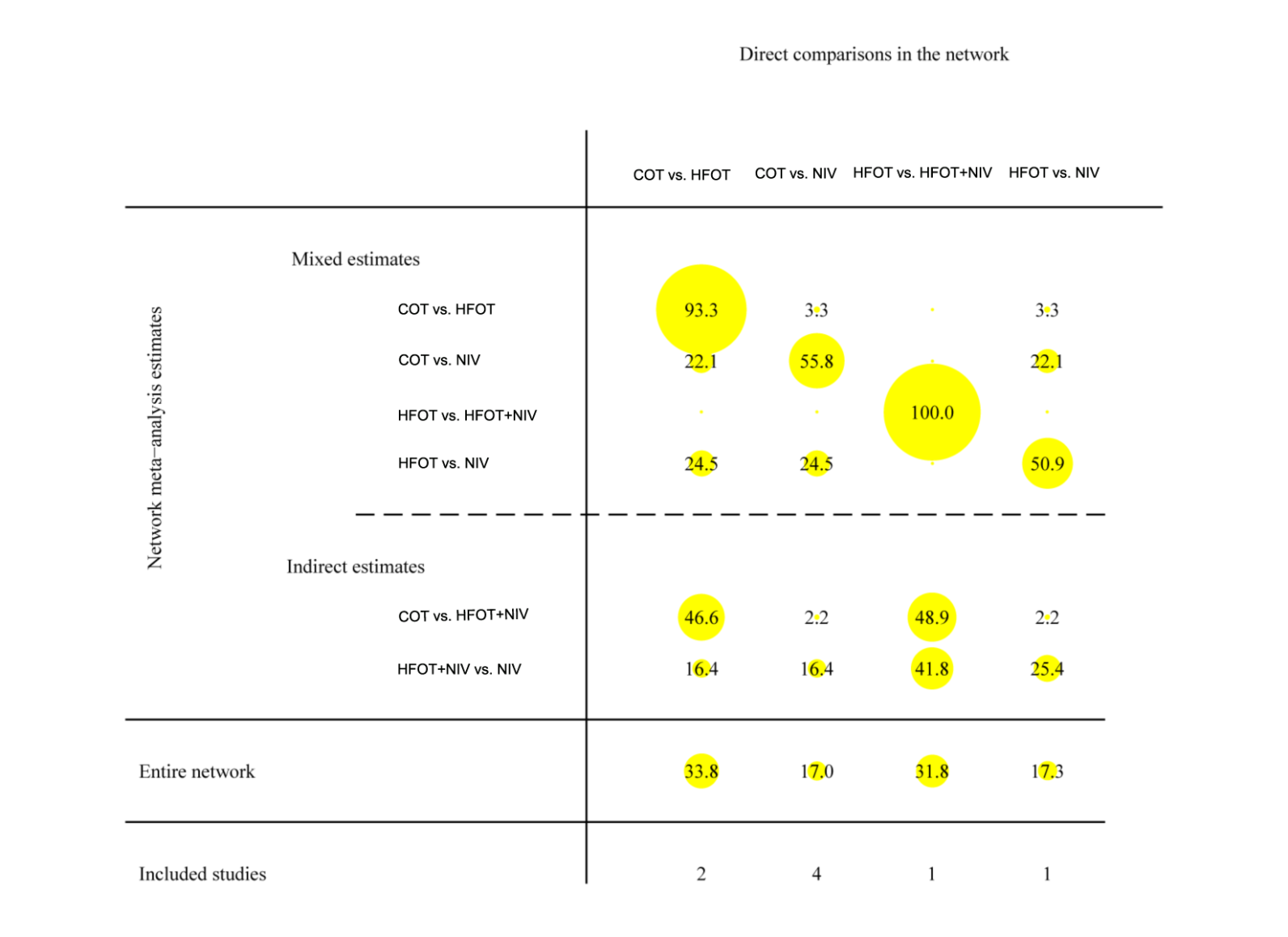

Supplement: Supplementary file 1 — Additional file 1. Detailed search strategies and supplementary tables and figures [file 13054_2020_3090_MOESM1_ESM.docx]
